# Inferring structure of cortical neuronal networks from activity data: A statistical physics approach

**DOI:** 10.1093/pnasnexus/pgae565

**Published:** 2024-12-19

**Authors:** Ho Fai Po, Akke Mats Houben, Anna-Christina Haeb, David Rhys Jenkins, Eric J Hill, H Rheinallt Parri, Jordi Soriano, David Saad

**Affiliations:** Department of Mathematics, Aston University, Birmingham B4 7ET, United Kingdom; Departament de Física de la Matèria Condensada, Universitat de Barcelona, Barcelona E-08028, Spain; Universitat de Barcelona Institute of Complex Systems (UBICS), Barcelona 08028, Spain; Departament de Física de la Matèria Condensada, Universitat de Barcelona, Barcelona E-08028, Spain; Universitat de Barcelona Institute of Complex Systems (UBICS), Barcelona 08028, Spain; College of Health and Life Sciences, Aston University, Birmingham B4 7ET, United Kingdom; Aston Institute for Membrane Excellence, Aston University, Birmingham B4 7ET, United Kingdom; College of Health and Life Sciences, Aston University, Birmingham B4 7ET, United Kingdom; Department of Chemistry, Loughborough University, Loughborough, Leicestershire LE11 3TU, United Kingdom; College of Health and Life Sciences, Aston University, Birmingham B4 7ET, United Kingdom; Aston Institute for Membrane Excellence, Aston University, Birmingham B4 7ET, United Kingdom; Departament de Física de la Matèria Condensada, Universitat de Barcelona, Barcelona E-08028, Spain; Universitat de Barcelona Institute of Complex Systems (UBICS), Barcelona 08028, Spain; Department of Mathematics, Aston University, Birmingham B4 7ET, United Kingdom

**Keywords:** biological neuronal networks inference, neuronal-type classification, kinetic Ising model, generalized maximum likelihood, expectation–maximization algorithms

## Abstract

Understanding the relation between cortical neuronal network structure and neuronal activity is a fundamental unresolved question in neuroscience, with implications to our understanding of the mechanism by which neuronal networks evolve over time, spontaneously or under stimulation. It requires a method for inferring the structure and composition of a network from neuronal activities. Tracking the evolution of networks and their changing functionality will provide invaluable insight into the occurrence of plasticity and the underlying learning process. We devise a probabilistic method for inferring the effective network structure by integrating techniques from Bayesian statistics, statistical physics, and principled machine learning. The method and resulting algorithm allow one to infer the effective network structure, identify the excitatory and inhibitory type of its constituents, and predict neuronal spiking activity by employing the inferred structure. We validate the method and algorithm’s performance using synthetic data, spontaneous activity of an in silico emulator, and realistic in vitro neuronal networks of modular and homogeneous connectivity, demonstrating excellent structure inference and activity prediction. We also show that our method outperforms commonly used existing methods for inferring neuronal network structure. Inferring the evolving effective structure of neuronal networks will provide new insight into the learning process due to stimulation in general and will facilitate the development of neuron-based circuits with computing capabilities.

**Significance Statement**

Inferring effective connectivity from firing patterns in neuronal networks is a long-standing problem, crucial for understanding how connectivity changes due to stimulation and plasticity occur. We introduce a probabilistic method to infer the excitatory/inhibitory type of neurons, links existence, and the effective coupling strengths in cortical neuronal networks from neuronal spiking recordings. We validate the efficacy of our method using both in silico and in vitro experiments, demonstrating that the effective structure revealed agrees with the true values and the key experimental observable such as modular organization. Additionally, the predicted neuronal activity using the inferred structures aligns with the observed data. The method will impact significantly on both fundamental studies in neuroscience and the development of neuron-based computing devices.

## Introduction

Revealing how cortical neuronal networks connectivity evolves in time, spontaneously or under stimulation, is a foundational question in neuroscience, in particular, understanding how cortical neurons learn from repeated stimulation through changes in topology and synaptic strengths (1–7). While there are many tools for investigating macroscopic brain activities and changes, such as functional magnetic resonance imaging (fMRI), magnetoencephalography (MEG), and electroencephalography (EEG) (8–10), investigating the microscopic changes which occur in neuronal tissues noninvasively remains a challenge. While in vivo interrogation of neuronal networks at the microscopic level remains difficult, in vitro techniques such as multielectrode array (MEA) (11) and calcium imaging (12, 13) facilitate the monitoring and stimulation of neuronal tissues at cellular resolution and open the way to greater understanding of the learning process. New developments in the application of neuron-based circuits with computing capabilities make the need to understand the exact relationship between learning and stimulation more urgent and relevant (14–17).

Machine learning (ML) and artificial intelligence (AI) play an increasingly crucial role in our daily lives. However, training ML and AI systems require unsustainable computing power and energy consumption (18) and mostly lack the ability to adapt their structure in response to a changing situation. This has given rise to the search for alternative computing paradigms, in particular, the emerging field of biological computation, which aims at employing human neuronal networks (hNNs) as processing units in biological computing devices. These developments, such as using cortical brain organoids for nonlinear curve prediction (19) and employing cortical neuronal networks for decision-making in simulated gaming environments (20), have recently drawn the attention of both researchers and the general public. These breakthroughs point to the immense potential of hNNs in biological machine learning. The common belief is that plasticity and learning occur through appropriate stimulation in neuronal-based computing devices, such that both network topology and synaptic strengths evolve to structures that can carry out specific data/stimulation-driven tasks. However, the tools needed to investigate the evolving structure and support the understanding of how these hNNs-based devices operate are currently lacking. Hence, it is crucial to develop a principled inference tool that can reveal the effective cortical network structure, to better comprehend the mechanism that gives rise to task learning from stimulation.

Various methods have been employed to infer the effective neuronal network from its firing patterns. Commonly used techniques include generalized transfer entropy (GTE) (21), dynamic causal modeling, Granger causality (22), maximum entropy model (23), and generalized linear model (24). However, these methods have significant limitations. They can only measure directional causation between neurons and identify the existence of an effective connection by setting an appropriate but somewhat arbitrary threshold. These methods cannot find the excitatory or inhibitory type of neurons without manipulating the network through stimulation or channel blocking (21, 25), nor can they determine the model’s effective synaptic strengths. As such, they are less suitable for studies requiring long-term monitoring and careful consideration of stimulation protocols as they may potentially affect network development as observed in the training of cortical neurons-based learning machines (19, 20).

Moreover, these methods often overlook the activities of nearby neurons and fail to capture interactions between multiple neurons, resulting in inaccurate inference. Additionally, methods such as GTE do not provide a probabilistic model for neuronal activity, making it difficult to predict or reproduce network activity using the inferred effective connectivity structure for validation or further investigations.

To fill these gaps, here we advocate mapping neuronal activities onto the kinetic Ising model of statistical physics as they share some common features, such as binary state of activity, unidirectional nonequilibrium and nonlinear dynamics, and multineuron interactions. Mapping neuronal activities onto the kinetic Ising model facilitates the inference of interneuron interactions (26, 27). Yet, inferring the kinetic Ising model structure and properties is challenging, and probabilistic methods have been developed in the statistical physics community for inferring the underlying directional interaction strengths from observation sequences (28–30). However, most methods have been derived for a simple model, where coupling strengths are Gaussian distributed with mean zero and small variance, which does not hold in biological neuronal networks, with the exception of (31) under specific conditions.^a^ Thus, a principled probabilistic method that can identify connectivity, synaptic strengths, and the excitatory/inhibitory type of each neuron is required.

In this paper, we introduce an algorithm that combines models from statistical physics, Bayesian inference, and probabilistic machine learning to infer the effective architecture of biological neuronal networks from firing patterns. Our proposed algorithm overcomes some of the limitations of existing methods and infers not only the effective connectivity from neuronal firing but also the neuronal characteristics (inhibitory/excitatory) and the existence of connections. Furthermore, unlike conventional methods, our algorithm provides a probabilistic model that facilitates the simulation of neuronal activities using the inferred architecture, which can be used for structure validation, prediction, and further investigations. We evaluate the performance of our algorithm using synthetically generated data, in silico neuronal network emulator data, and calcium imaging recordings of real in vitro cortical networks with patterned (32) and unpatterned substrates, demonstrating excellent agreement between the effective inferred model and the corresponding data.

## Model

### Kinetic Ising model

The kinetic Ising model (33) in statistical physics studies the activity of spins in a system with asymmetric coupling strengths. Sharing the common feature that spin (neuron) configuration is influenced by directional coupling (synaptic) strengths and the state of its neighbors, the kinetic Ising model is suitable for describing neuronal spiking activities. Here, we map the binary neuronal spiking activity onto the kinetic Ising model, which is a discrete-time nonequilibrium probabilistic structure. Consider a system of *N* neurons within an interacting neuronal network. We denote a discrete variable $s_{i}^{t}=\pm 1$ when neuron *i* is spiking or silent at time step *t*, respectively, for i=1,…,N. Previous works (21) suggest that considering interactions across multiple time intervals has minimal effect on the inference, so we define the transitional probability of neuron *i* at time *t*, given the neuronal activities at time t−1, as


(1) $$P\left(s_{i}^{t}\;|\;s^{t-1},J,H_{i}\right)=\frac{\exp\;\left[\left(H_{i}+\underset{\;j}{\sum }\;J_{ij}s_{\;j}^{t-1}\right)s_{i}^{t}\right]}{2\cosh\;\left(H_{i}+\sum_{\;j}J_{ij}s_{\;j}^{t-1}\right)},$$

where $J=\{J_{ij}{\}}_{ij}$ and $J_{ij}$ denotes the synaptic strength from *j* to *i*, and $H_{i}$ denotes the external local field acting on neuron *i*, which can be interpreted as the activeness of *i* when no signal is received from its neighbors. A positive (negative) $J_{ij}$ represents the excitatory (inhibitory) strength of a signal sent from neuron *j* to neuron *i* when *j* spikes, while $J_{ij}=0$ indicates that neuron *j* is not effectively connected to *i*. Since the activities of neurons at time *t* depend only on activities in the previous time step and hence do not exhibit explicit same-bin interdependence, the probability of activities for all neurons is given by $P(s^{t}\;|\;s^{t-1},J,H)=\prod_{i}P(s_{i}^{t}\;|\;s^{t-1},J,H_{i})$. In order to infer the neuronal type and effective link existence, we introduce two sets of latent variables, $z_{j}=\pm 1$ representing the excitatory and inhibitory type of neuron *j*, respectively; and $\phi_{ij}=\{1,0\}$ indicating whether *j* is connected to *i* or not, respectively. One of the limitations of the kinetic Ising model is that it does not consider longer synaptic time delays, but as indicated in a number of studies (2, 21, 34), their influence is much weaker than that of single time step delays.

### Adapting Ising model to living neuronal networks

Based on the mathematical model defined, we introduce prior distributions to align with real-world neuronal network properties. For clarity, we use p(⋅) to denote prior distributions for the corresponding variables, while P(⋅) denotes the probability more generally. We define


(2) $$p(z_{\;j})=\gamma \delta_{z_{\;j},+1}+\left(1-\gamma \right)\delta_{z_{\;j},-1};$$

(3) $$p\left(\phi_{ij}\;|\;a\right)=\delta_{\phi_{ij},1}\theta_{ij}e^{-al_{ij}}+\delta_{\phi_{ij},0}\left(1-\theta_{ij}e^{-al_{ij}}\right);$$

(4) $$p\left(J_{ij}\right)=\sum_{z_{\;j}}\sum_{\phi_{ij}}p\left(J_{ij}\;|\;\phi_{ij},z_{\;j}\right)p\left(\phi_{ij}\right)p\left(z_{\;j}\right);$$

(5) $$p\left(H_{i}\right)=\frac{e^{-\frac{(H_{i}-\mu_{H}{)}^{2}}{2v_{H}}}}{\sqrt{2\pi v_{H}}},$$

where γ∈[0,1] represents the proportion of excitatory neurons in the network; $\theta_{ij}$ is the prior probability for the existence of a link from neuron *j* to neuron *i*, which can be adjusted for convergence assistance in networks with predefined or dictated connectivity (32, 35), or set to 1 otherwise; $a\in R^{+}$ is a controlling parameter that accounts for the decay in connection probability; and $l_{ij}=l_{\;ji}$ the Euclidean distance between *i* and *j*, thus $e^{-al_{ij}}$ reflects the exponential decay in connection probability with distance; $\mu_{H}$ and $v_{H}\in R$ represent the mean and variance of the distribution for H, reflecting the distribution of neuron inherent activeness. The distribution of $J_{ij}$ comprises a mixture of distributions since it is conditioned on whether there exists a link between two neurons and whether the type of the interaction is inhibitory or excitatory; the different cases may be characterized by different parameters. We define the conditional probabilities $p(J_{ij}\;|\;0,z_{j})=\delta (J_{ij})\approx N(0,\epsilon )$ for small ϵ (disconnected case) and $p(J_{ij}\;|\;1,z_{j})=1_{z_{j}J_{ij}>0}e^{-(\ln\;z_{j}J_{ij}-\mu_{J}^{z_{j}}{)}^{2}/(2v_{J}^{z_{j}})}/\sqrt{2\pi v_{J}^{z_{j}}}$ for $\mu_{J}^{z_{j}},v_{J}^{z_{j}}\in R^{+}$ and $z_{j}=\pm 1$, where 1 is an indicator function ensuring all connections from *j* are of the same sign, and $z_{j}J_{ij}$ follows a log-normal distribution to ensure the probability of coupling strengths of inhibitory or excitatory neurons sum to 1. The compact notation merely deals with the positive (excitatory) and negative (inhibitory) interaction strengths within the same expression by allowing for different distribution characteristics. For brevity, we denote $\rho =\{\gamma ,a,\mu_{H},v_{H},\mu_{J}^{\pm },v_{J}^{\pm }\}$ as the set of all defined hyperparameters (parameters that control the distributions of J and H). By combining the distributions we define the evidence function as


(6) lnP(s|J,H,ρ)+lnp(H|ρ)+lnp(J|ρ).

In principle, the related probability of Eq. 6 should be integrated over J and H to obtain the marginalized probability P(s|ρ) but is approximated at the peak of the posterior (“Maximum A Posteriori,” MAP) (36).

### Connectivity network inference

We extend the kinetic Ising model by introducing two sets of latent variables and prior distributions for all variables to align with the nature of realistic biological neuronal networks. The Methods section and the Supplementary material provide details on how the (latent) variables and hyperparameters can be evaluated in a principled way, enabling us to interpret the neuronal type from $z_{j}$, link existence through $\phi_{ij}$, and effective synaptic strength from $J_{ij}$. In general, we are applying the generalized maximum-likelihood (GML) approach (whereby parameters and hyperparameters are iteratively determined, also termed the evidence procedure) and the variational expectation–maximization (EM) algorithm (36) to infer J, H, and ρ.

## Results

To validate the efficacy of our model and inference algorithm, we perform tests using three types of data: synthetic data generated by the kinetic Ising model, in silico data from computational models, and in vitro data from biological cortical neuronal activity recordings.

In the Supplementary material, we discuss and examine the properties of different algorithms for kinetic Ising model inference, comparing them with our proposed method in detail, and show that the proposed method performs significantly better than the naïve mean-field and maximum-likelihood approaches. In our GML inference for the kinetic Ising model, coupling strengths are not zero-mean and of small variance, and more importantly, the method allows one to infer the existence of connections between neurons. Here, we focus on the results obtained from the in silico model with patterned substrates as well as the two realistic in vitro cortical neuronal activities. One of these in vitro activities is of a homogeneous network with potassium stimulation, while the other focuses on spontaneous activities in a neuronal network with patterned substrates.

###  

#### In silico experimental data

**Fig. 1.**
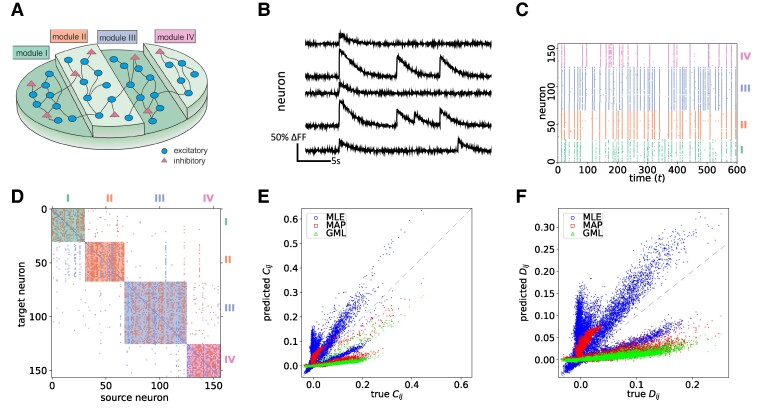
A) A three-dimensional sketch illustrating the in silico experimental setup. The neurons (N=156) are lying on stripes (modules I to IV) of patterned substrates, which suppress cross-connections between different stripes. B) Example in silico traces of five neurons. C) The raster plot displays neuronal activity inferred from the in silico traces, with each neuron’s color corresponding to the module it belongs to. D) The inferred structure J of the in silico model obtained using GML and represented by a connectivity matrix. The background color is a mixture of two colors, with each color corresponding to the module of the source and target neurons. Each entry corresponds to the coupling strength $J_{ij}$. A positive (negative) strength colored in red (blue) refers to excitatory (inhibitory) signals sent from source (*i*) to target (*j*) neurons. E) The predicted equal time covariance C against the true values evaluated from data. F) The predicted delayed time covariance D against the true values evaluated from data.

Since validating the accuracy of neuronal-type classification and link existence is very costly and extremely difficult for in vitro experiments, we first test our algorithm on emulated in silico data since ground-truth topology is known. We generate an in silico neuronal network in which the culture is lying over a striped topographical patterned substrate (32) as illustrated in Fig. 1A. The patterned substrate facilitates high connection density between neurons located on the same stripe but allows for lower connection density across stripes, which is an interesting feature that can be expressed and tested using our method. Spontaneous neuronal activity is then generated on this network. We note that connectivity within a stripe is so strong that each one effectually shapes a “module” of highly interacting neurons. The detailed methodology of how the data are generated is described in the Methods section.

Figure 1B shows representative fluorescence traces of spontaneous neuronal activity generated in silico, and Fig. 1C shows the raster plot of activity for the entire network, with colors indicating the respective module neurons belong to. Using the activity as input, we infer the effective structure J using the Maximum-Likelihood Estimator (MLE), MAP, and GML. Their methodology is described in detail in the Methods section and Supplementary material. For the prior distributions imposed in MAP and the evidence approximation, we first fix the hyperparameter a=0.1, the fraction of excitatory neurons, γ=0.8 and $\theta_{ij}=0.5^{m_{d}}$, where $m_{d}$ is the number of stripes that separate neurons *i* and *j*. The values employed for the other hyperparameters used both for MAP inference and as initial values for the GML inference, are obtained using J and H inferred by MLE. The value of *a* reflects the fact that neurons are less likely to be connected if they are far away from each other; *γ*, the ratio between excitatory and inhibitory neurons is already statistically known; while for $\theta_{ij}$, we exploit available information about the physical structure and the value of 0.5 is chosen arbitrarily. We tested the performance of the inference methods using other values of *a*, *γ*, and $\theta_{ij}$ and the results are similar. This is because when the number of samples is large enough, the effect of the priors is suppressed. Table S1 specifies all parameters, their type, description, and the values they acquire for clarity.

The inferred connectivity matrix of J using GML is shown in Fig. 1D, where the background mask is the mixture of colors corresponding to the source and target neurons, while inhibitory and excitatory transmissions are colored in blue and red, respectively. We note that the diagonal entries are mostly inhibitory; this does not necessarily correspond to physical connections but may reflect the fact that neurons are less likely to spike again after firing due to their refractory period. Another key feature is that except for the diagonal, signals in the same column share the same state, which corresponds to the inferred excitatory/inhibitory type of the neurons; this is validated by comparing the inference results to the true in silico model.

The most appropriate measures of success when contrasting inferred (“infer”) and ground-truth (“true”) topologies are the positive predictive value (PPV) and negative predictive value (NPV), or precision of neuronal type $P(z_{\mathrm{true}}\;|\;z_{\mathrm{infer}})$, compared with the prior-based random guess, where excitatory type is the positive case and inhibitory the negative one, as Table 1 shows. While being less relevant due to the biased nature of the variables, the true positive rate (TPR, sensitivity) and the true negative rate (TNR, specificity), $P(z_{\mathrm{infer}}=+/-\;|\;z_{\mathrm{true}}=+/-)$ are also presented alongside the random guess, for completeness. Overall predictive performance is summed over both cases with the respective probabilities. Notably, no existing method can infer the excitatory and inhibitory type of neurons from single spontaneous activity recordings without interference, such as channel blocking. With a significant improvement over a prior-based random guess, our method identifies well the individual neuronal type.

**Table 1.** Success measures in identifying neuron type, TPR (sensitivity), TNR (specificity), and PPV of the in silico model study.

Measure	TPR/TNR	PPV/NPV	Random
Excitatory	0.88	0.96	0.8
Inhibitory	0.84	0.64	0.2
Overall	n/a	0.87	0.68

**Fig. 2.**
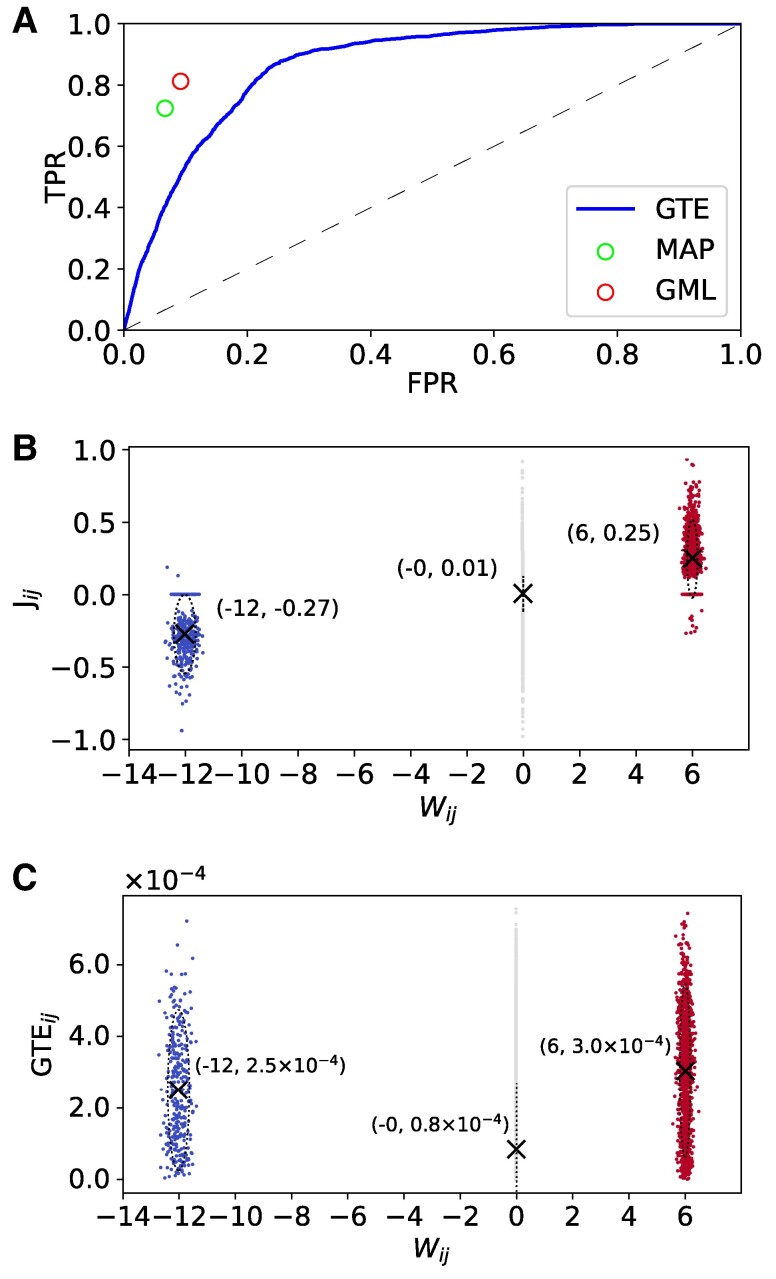
A) The ROC curve plotting the TPR against the false positive rate (FPR) of identifying effective links for in silico experiments using GTE (continuous blue line). The TPR and TFR using our MAP and GML are marked as green and red nodes respectively. The dashed diagonal line in A refers to a random guess in this case. B and C) The scatter plot of inferred coupling strength $J_{ij}$ and GTE against the true synaptic strength of the in silico model, respectively. The inhibitory, excitatory, and nonexisting links are colored in blue, red, and gray, respectively.

Figure 1D reveals a strong clustering effect for connectivity within modules, indicating that two neurons within the same module have a higher probability of being connected compared with two neurons from different modules. This suggests that the GML captures the structure of the topographical substrate. We then test the performance of inferring existing links using MAP, GML, and GTE (21). Since GTE is based on an assigned transfer entropy threshold value for identifying links, we plot the complete receiver operating characteristic (ROC) curve for all threshold values, of TPR against FPR of identifying nonzero links as shown in Fig. 2A. The TPR and FPR of finding nonzero links using GML are 54% and 2.6%, respectively, and 57% and 3.3% for MAP, as indicated by the red and green circles, respectively. These values exceed the ROC curve generated by GTE, indicating that our method offers a higher TPR than GTE at the same FPR level, or a lower FPR at the same TPR level. We remark that, although MAP has a higher TPR than GML, its FPR is also higher. Additionally, we observe that the difference between MAP and GTE at the same FPR is lower than that of GML, which emphasizes the importance of hyperparameter optimization. It is essential to point out that our approaches detect link existence in a principled manner, eliminating the need for heuristic threshold decisions, and rendering the inference results more reliable.

A key feature that our method offers is the inference of the effective synaptic (coupling) strength between neurons, which is crucial for understanding the learning process in both neuroscience and cortical neuron-based learning machines. For example, observing changes in synaptic strengths over extended periods allows one to study neuronal network plasticity due to stimulation and facilitates the design of efficient stimulation protocols in cortical neurons-based learning devices. We note that the neuronal spiking mechanism of the in silico emulator follows the Izhikevich model (37), which is distinct from the kinetic Ising model so that there is no direct mapping between the inferred ($J_{ij}$) and true synaptic strength ($W_{ij}$, actual values used in emulator simulations). To compare the capability of our method with existing techniques, we plot the GML inferred weights $J_{ij}$ and GTE values of each link against the true synaptic strengths $W_{ij}$ in Fig. 2B and C, respectively. In the true model, the mean value of the inhibitory synaptic strength is −12, considerably higher than the excitatory synaptic strength’s mean value of 6. GTE as a specific manifestation of transfer entropy only offers nonnegative scores and therefore cannot directly distinguish inhibitory and excitatory links.

Figure 2C shows the mean value of GTE in the inhibitory group to be $2.6\times 10^{-4}$, which is lower than the value in the excitatory group, $3\times 10^{-4}$—contradicting the true values. On the other hand, as Fig. 2B shows, the mean value of the inferred $J_{ij}$ for the inhibitory connections is −0.27, which is of higher magnitude than that of excitatory connections, 0.25. These results suggest that the inferred $J_{ij}$ using our method agrees with the true model synaptic strengths ($W_{ij}$). The GTE values also exhibit a significant variance compared with the magnitudes of the mean values. Notably, the mean value of the inferred $J_{ij}$ for the missing links is very close to zero with a small variance, indicating that although some of the links are classified as nonzero, incorrectly, the majority are overwhelmingly close to zero. This false positive classification may result from the Gaussian approximation of the delta function in the prior probability $p(J_{ij})$. This suggests that the inferred effective coupling strength using our GML method agrees with the true model.

Another advantage of our method is that one can adopt the inferred structure J and H for generating artificial data through Monte Carlo simulations to predict neuronal activity or validate how well the inferred structure describes the true model by comparing quantities of interest, such as the equal time covariance $C=\{\langle s_{i}^{t}s_{\;j}^{t}\rangle_{t}{\}}_{i,j}$, and the delayed time covariance $D=\{\langle s_{i}^{t}s_{\;j}^{t-1}\rangle_{t}{\}}_{i,j}$. Thus, we employed the inferred structures using MLE, MAP, and GML to generate artificial neuronal activity and evaluate the predicted equal time covariance C and delayed time covariance D to its true values, as Fig. 1E and F shows. We can see that the predicted C and D values closely align with the true values for all methods, suggesting that the kinetic Ising model effectively explains in silico neuronal activities.

Our GML approach demonstrates strong performance in inferring the neuronal types and link existence as well as the ability for activity prediction. Notably, by comparing the results between MLE and our GML approach, we observe that GML exhibits a gentler slope and deviates more from the perfect prediction manifested by the y=x line. However, our results still demonstrate a strong overall agreement. This difference may be due to the fact that MLE does not impose any restrictions on the choice of $J_{ij}$, aiming to provide the best possible description of the data. On the other hand, in GML, the prior probability $p(J_{ij}\;|\;0,z_{j})≊N(0,\epsilon )$ acts as a regularization term and constrain $J_{ij}$ to have the same sign for each fixed *j*. As a result, the inferred $J_{ij}$ values are lower than those obtained through MLE, leading to an underestimation of D. Nonetheless, this approach allows for accurate predictability of neuronal types and effective links, which is more important in neuroscience research.

The above results show that our GML approach performs very well on data generated by the in silico model with patterned substrates. In the Supplementary material, we study the performance of our GML approach on another in silico homogeneous neuronal network with no patterned substrates. We show that even in a homogeneous network, which is harder for structure inference, as there are fewer constraints for the solution space of J, our GML approach still performs very well. The results obtained by the GML for in silico data support the view that it is a highly suitable candidate for analyzing biological neuronal network data.

#### Sensitivity analysis of in silico experiments

To further investigate the performance of our algorithms in different scenarios, we performed a sensitivity analysis on in silico neuronal networks where synaptic strengths follow different distributions, varying the degree-connectivity from sparse to dense. Results for networks with patterned substrates are shown below, while results for homogeneous networks are provided in the Supplementary material.

We examine two different cases where the synaptic strengths in the in silico networks vary. Specifically, we assume that the synaptic strengths $W_{ij}$ follow a Gaussian distribution with mean $\Omega_{E}$ ($\Omega_{I}$) and variance 0.05, for excitatory (inhibitory) neurons. Starting with a network over stripe-patterned substrates, where there are no connections between neurons (i.e. all axon lengths are initially zero), we gradually increase the degree-connectivity by growing the axon lengths, as detailed in the Methods section. At different connectivity levels, we take snapshots of the network structure to generate neuronal activities. We perform the analysis on an in silico neuronal network over stripes patterned substrates, consisting of N=156 neurons, averaged over five samples. We study two cases: strong synaptic strengths with $\left(\Omega_{E},\Omega_{I})=(6,12\right)$ and weak couplings with $\left(\Omega_{E},\Omega_{I})=(4,8\right)$. Due to the high computational complexity of GML, we performed structural inference using MAP only for this sensitivity analysis.

**Fig. 3.**
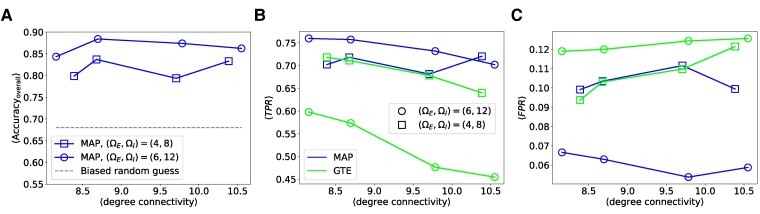
Sensitivity analysis of the performance of MAP and GTE on structural inference in in silico neuronal networks over stripe-patterned substrates with different synaptic weight distributions, varying over the average degree-connectivity. A) Average overall performance in neuronal-type classification, compared with a biased random guess. B) TPR of identifying effective links. The TPR of GTE is obtained from the ROC curve by fixing the FPR at the same value as MAP. C) FPR of identifying effective links. The FPR of GTE is obtained from the ROC curve by fixing the TPR at the same level as MAP. Results are averaged over five samples for a network of N=156 neurons.

As no existing method can perform neuronal-type classification, we compare our results with those from biased random guessing, as shown in Fig. 3A. The average overall accuracy of neuronal-type classification for both weak and strong synaptic strengths is significantly higher than random guessing, indicating strong performance. For effective connectivity identification, we compare our method with GTE. As discussed, GTE provides an ROC curve, not specific TPR or FPR values. To facilitate comparison, we fix the FPR for GTE at the same level as MAP to obtain the TPR value, and vice versa. Figure 3B and C shows the resulting TPR and FPR values, respectively. For the strong synaptic weights case, our method achieves higher TPR and lower FPR than GTE, outperforming GTE in both metrics. When synaptic weights are weak, our method shows only a slightly higher TPR and slightly lower FPR than GTE. This is due to weaker spiking activity and lower correlations between neurons, making structural inference more challenging. While MAP considers interactions among multiple neurons, which requires more data, GTE focuses on pairwise interactions only, allowing for quicker inference with less data. Nevertheless, our method outperforms GTE in both cases.

**Fig. 4.**
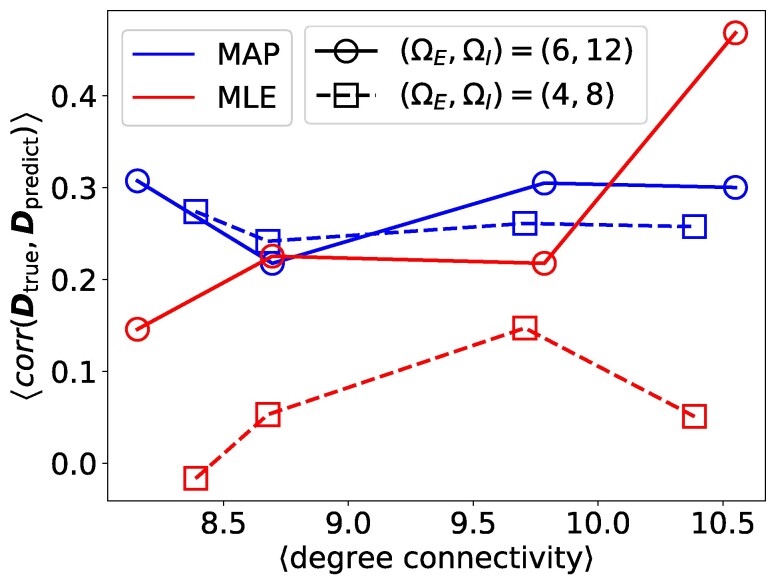
Sensitivity analysis of the predictability performance on in silico neuronal networks with stripe-patterned substrates using MAP and MLE. The average correlation between predicted and true delayed time covariance D, for strong and weak synaptic strengths, as a function of the average degree-connectivity. Results are averaged over five samples for a network of N=156 neurons.

To further assess the predictive capability of our method, we generated predicted activities using Monte Carlo simulations based on the structures inferred by MAP and MLE for each sample, and compared them with the true activities by measuring the correlation between predicted and true delayed time covariances D as shown in Fig. 4. It is worthwhile noting that correlation is a more appropriate success measure than other metrics like root-mean-squared error, since it measures the alignment between the true and predicted D. As shown in Fig. 4, for strong synaptic strengths the average correlation between the predicted and true values of D using MAP is higher than that of MLE when the average degree-connectivity is low. For weak synaptic strengths, MAP performs significantly better than MLE across all degree-connectivities. Although MLE aims to find the optimal configuration for the couplings without restrictions, when the degree-connectivity is low or synaptic strengths are weak, neurons are relatively inactive, making it difficult for MLE to find good solutions. The restrictions in MAP make it easier to find a solution, yielding better activity predictions.

Our sensitivity analysis demonstrates that our algorithms do not only work effectively in single instances but also perform well across various connectivity and synaptic strength scenarios.

#### Experimental validation—in vitro network with patterned substrates

**Fig. 5.**
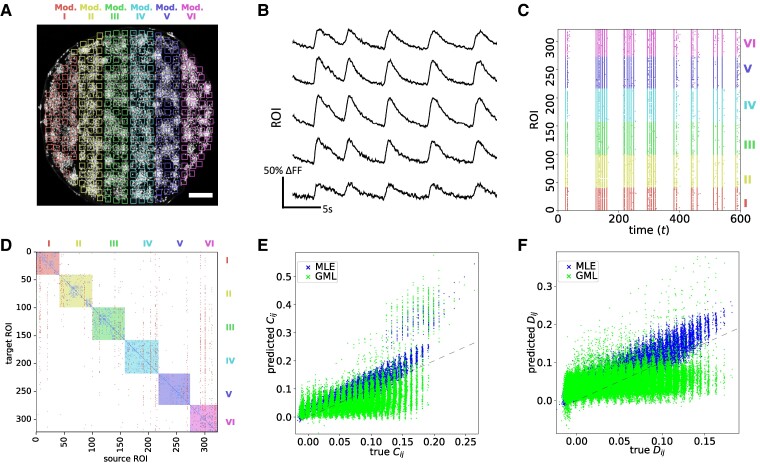
A) The studied rat cortical neuronal network over striped a topographical pattern 2 mm in diameter, grouped into 400 regions of interest (ROIs). ROIs within different stripes are shown as squares with different colors. The scale bar of 200 μm is displayed in white in the figure. B) Example in vitro calcium imaging traces of five ROIs. C) The raster plot displays neuronal activity inferred from the calcium traces, with each ROI’s color corresponding to the module it belongs to. D) The connectivity matrix of the effective structure inferred from the in vitro neuronal activity, using the proposed GML method. Each entry shows the coupling strength of the connections, a negative (positive) strength represents an inhibitory (excitatory) connection and is colored in blue (red). The background shade of the plot is the module that the ROI belongs to, with mixed colors where ROIs from two different stripes are connected. A strong clustering effect suggests a dense connectivity of ROIs within stripes and sparse connectivity across stripes. E and F) The predicted equal time covariance C and delayed time covariance D matrices, plotted against the true values evaluated from the data, respectively.

Having validated the efficacy of our GML approach to both synthetic data (see Supplementary material) and emulator data, we now apply it to an in vitro rat cortical neuronal network grown on topographically patterned substrates. Data consisted of neuronal activity recording from calcium fluorescence imaging, processed to consider regions of interest (ROIs) as nodes in the neuronal network, as shown in Fig. 5A. Details of data acquisition and analysis are provided in the Methods section.

Using this experimental data, with representative traces in Fig. 5B and complete raster plot in Fig. 5C, we infer the effective structure and recreate the neuronal activity for validation. Similar to the in silico case, we group ROIs into modules according to the stripes patterning (Fig. 5A); the inferred coupling strengths are visualized in the connectivity matrix shown in Fig. 5D. The background colors indicate which module the source ROIs belong to. Entries colored in red (blue) indicate effective excitatory (inhibitory) connections from source (*i*) to target (*j*) ROIs. We observe dense connectivity between ROIs from the same stripe and sparse connections across stripes. Furthermore, the likelihood of connection decreases as the distance between ROIs increases. This agrees with the biological understanding that long-range connections are rare to minimize wiring cost, so that neurons preferentially connect to their neighbors, as observed in previous studies using similar patterned substrates (32).

While conventional approaches often yield sets of scores, like GTE, they lack a direct measure of estimation quality. Our probabilistic model-based inference, however, allows for the validation of inferred effective structures by reproducing neuronal activity using Monte Carlo simulation. We thus simulate activity using the estimated reconstructed effective network parameters J and H and compare them with the experimental values, evaluating afterward the equal and delayed time covariance matrices C and D, as shown in Fig. 5E and F, respectively. Most of the predicted values are aligned with the dashed lines (exact match), meaning a good-quality prediction. However, one can see that for both C and D, some of the predicted values are very close to zero. This can be attributed to false positive errors generated due to the restrictions imposed on individual ROIs, such as having the same characteristics, e.g. being either excitatory or inhibitory and sharing the same synaptic connection sign. More realistically, within a single ROI, there might be neurons of different types, leading to some connections being erroneously decimated to zero. We anticipate this problem to be mitigated when smaller ROI sizes are considered and longer calcium recordings are used, which will be discussed in the next section.

In general, the GML inference method shows excellent agreement between the inferred effective network and the underlying biological structure, with predicted activities fitting well with the real data.

#### Altered in vitro homogeneous network structure by modifying neuronal activity

To validate the algorithm further in a scenario of changing synaptic strengths, we implement experiments that neuronal connectivity is altered through plasticity, which is modulated via chemical stimulation.

Arguably, the simplest way to understand how neuronal network plasticity occurs due to stimulation is to compare the effective network structures of a neuronal network before and after stimulation. We apply our method to an in vitro primary homogeneous neuronal network comparing the situation in control and following exposure to elevated potassium chloride (KCl) stimulation, with the aim of depolarizing neurons and therefore increasing network activity. Neuronal activity data was also collected through calcium imaging, although here each ROI includes one neuron only. The video of the calcium imaging recording is available from (38). The details of data generation are provided in the Methods section.

**Fig. 6.**
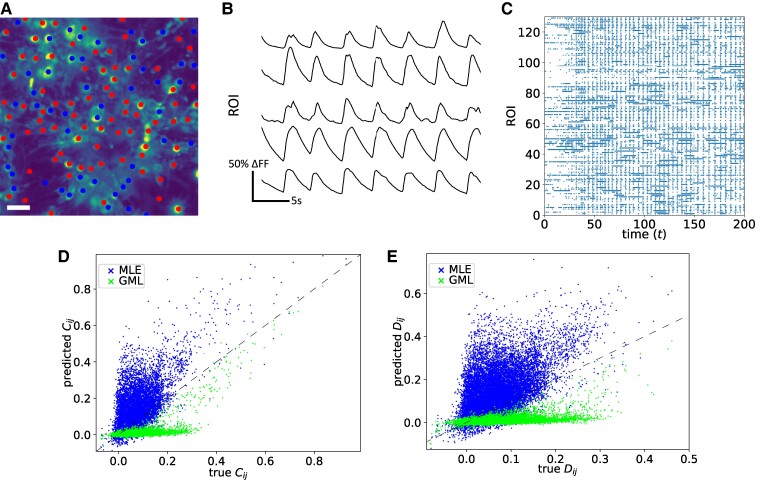
A) The in vitro primary cortical homogeneous neuronal network analyzed, grouped into 131 regions of interest (ROIs). The red and blue dots correspond to the regions of interest (ROIs) that are classified as excitatory or inhibitory, respectively, where each includes one neuron only. The scale bar of 25 μm is displayed in white in the figure. B) Example in vitro calcium imaging traces of five ROIs. C) The raster plot displays neuronal activity inferred from the calcium traces of each ROI. D and E) The predicted equal and delayed time covariance matrices generated by the inferred structure using Monte Carlo simulation, C and D, respectively, plotted against the true values evaluated from the data.

We infer the effective structure using the neuronal activity data as input, with representative traces in Fig. 6B and complete raster plot in Fig. 6C, and then recreate the activity measures for validation. We first identify the neuronal type (of single neuron ROIs) as excitatory (red) and inhibitory (blue) as shown in Fig. 6A.

In particular, we simulate activity using the inferred J and H, then evaluate the equal and delayed time covariance C and D and compare them with the true covariance values, as shown in Fig. 6D and E, respectively. Similar to the case of the patterned substrate, some predicted values are very close to zero for both C and D. However, compared with Fig. 5E and F, we see that the predicted values align better with the dashed line, suggesting a more accurate prediction capability. We note that data shown in Fig. 5 and 6 are both acquired using calcium imaging approaches, but the resolution of the experiment in Fig. 6 enables the identification of single neuronal elements. This suggests that by significantly reducing the size of each ROI to consist of a single neuron, one can reduce errors with respect to multiple neurons ROIs.

In this section, we have studied two in vitro experiments providing different scopes. In the experiment with patterned substrates, we have studied a completely extended network; whereas in the experiment with a homogeneous network, we have studied neuronal activity modulated by chemical stimulation. While the samples and the experimental conditions are different, it is interesting to see that both covariance matrices show a much higher value under stimulation as the KCl stimulation affects the activity across the sample. A detailed study comparing the neuronal structure and synaptic weights before and after stimulation is underway and is beyond the scope of this work. Our results demonstrate that our methods do not only work effectively on synthetic in silico data but also perform well on more realistic neuronal networks.

## Discussion

Cortical neuronal network inference has long been an open question in neuroscience and is crucial for understanding the underlying mechanisms and properties of neuronal systems. Neuronal cultures are regarded as a promising living model to investigate a broad spectrum of technological challenges, from biologically inspired AI (14, 20) to efficient design of treatments for neurological disorders (39). Since the structural blueprint of neuronal connections is not easily accessible in a culture, nor their excitatory/inhibitory type, indirect techniques to infer such a blueprint have jumped into the front-line of computational neuroscience.

Here we introduced a novel and probabilistic algorithm based on statistical physics and Bayesian techniques for the effective structural inference of biological neuronal networks from activity data. The algorithm can not only infer the effective synaptic strengths between neurons but, more importantly, can identify the excitatory and inhibitory type of neurons as well as the effective connections between them in a probablistic way, a capability that no other existing method can achieve. This is used in a principled way from single spontaneous recordings without additional interference to the culture such as stimulation. This capability goes beyond what most existing state-of-the-art methods can offer. Through synthetic, in silico and realistic in vitro experiments, we demonstrate that our algorithm: (i) outperforms existing methods in both synaptic strength inference and effective connections identification; (ii) achieves high accuracy in neuronal-type classification; (iii) exhibits good reproducibility in the inferred structure, justifying the reliability of the algorithm. In the Supplementary material, we also show that our algorithm provides good inference results also in the absence of patterned substrates, in both in silico and in vitro studies.

The dynamics and functioning of neuronal networks are in large part determined by their connectivity and their evolving synaptic strengths. As such, the method is expected to have a direct impact on neuroscience, cortical neuron-based computing devices, and many other related biological and medical areas. For instance, studying the changes in the effective structure of neuronal cultures over time can lead to new theoretical understandings of how plasticity takes place in response to stimuli. Additionally, gaining insight into information processing and propagation in neuronal networks could greatly impact the development of artificial neuronal networks and neuromorphic computing, e.g. by understanding the importance of the ratio between excitatory and inhibitory neurons on functionality.

Revealing precise information about effective structure and neuronal types is essential for developing biological machine learning as it helps one to accurately represent the network dynamics, make predictions, and test hypotheses. Most importantly, it is critical for the design of stimulation learning protocols. Interdisciplinary research in this direction is underway.

## Methods

### Preprocessing and optimal time bin determination

Since the mathematical model we introduce is based on discrete-time steps, one needs to binarize the neuronal activity before carrying out the inference process. We note that the determination of the time bin *τ* is crucial for inference as the binarized firing patterns can vary significantly with *τ*. For instance, the delayed time covariance between neurons can vanish if *τ* is either too large or small. To address this, we employed an information theory-based method to identify the optimal time bin size $\tau^{*}$ that optimizes the total mutual information of the system (26, 27). The optimal bin size $\tau^{*}$ is given by


(7) $$\tau^{*}=\underset{\tau }{\text{argmax}}\;\left[\left(\frac{T}{\tau }-1\right)\sum_{i\neq j}I_{\tau }\left(s_{i},s_{\;j}\right)\right],$$

where $I_{\tau }(s_{i},s_{\;j})$ is the mutual information between $s_{i}^{t}$ and $s_{\;j}^{t-1}$. The idea of mutual information is straightforward: $I_{\tau }(s_{i},s_{\;j})$ measures the discrepancy between the joint probability $P(s_{i}^{t},s_{\;j}^{t-1})$ and the factorized probability $P(s_{i}^{t})P(s_{\;j}^{t-1})$ where activity of *i* and delayed activity of *j* are assumed to be independent. Thus, a higher $I_{\tau }(s_{i},s_{\;j})$ suggests stronger correlation between neurons *i* and *j*. Thus, $\tau^{*}$ maximizes the total mutual information, indicating that the neurons are least likely to be independent of each other and more information about their co-dependence can be extracted. Intuitively, $\tau^{*}$ can be understood as the average effective reaction time, starting from when a neuron spikes, the spike is transmitted through the synapse and until the target neuron responds. Using $\tau^{*}$ evaluated in Eq. 7 to discretize the neuron firing times into distinct time steps that provide the observed data s, which is then ready for the inference process.

### Inference algorithm

Unlike the basic kinetic Ising model of statistical physics, inferring the effective structure from neuronal activities faces two major challenges: (i) J is not Gaussian with small variance due to the existence of different neuron types; (ii) the connectivity between neurons is affected by multiple factors, including neuronal distance and the patterned substrates (e.g. PDMS stamps). These factors make it difficult to successfully adapt established approaches (28, 29, 40–45) directly to this problem.

To tackle these challenges based on the defined mathematical model, we utilize the generalized maximum-likelihood (GML) (36) technique, also known as evidence approximation and MAP estimation. This combination enables us to jointly infer optimal hyperparameters, latent variables, and the effective network structure in a principled manner. While the detailed derivation is available in the Supplementary material, we provide a general overview of the method here.

**Fig. 7.**
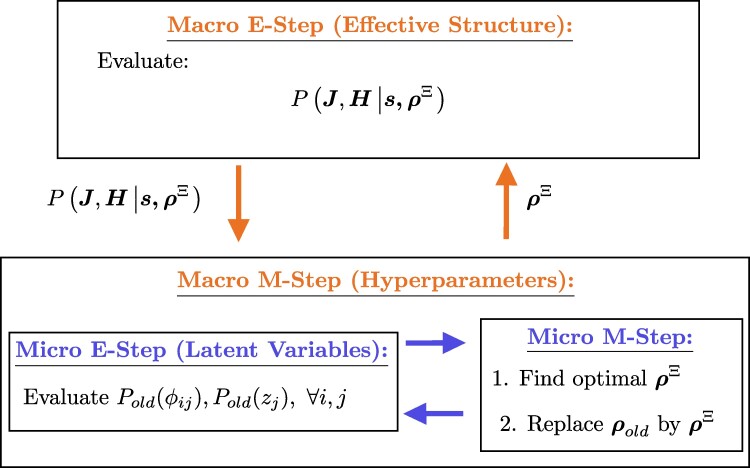
A sketch of the proposed GML-based approximation algorithm. The effective connectivity and the optimal hyperparameters are jointly evaluated by incorporating two applications of the EM algorithm. The posterior distributions of J and H are evaluated in the macro E step, while the hyperparameters values are evaluated in the macro M step, which has an internal nested EM algorithm of its own, representing the interplay between the latent variable values and their distributions. In the micro EM algorithm: the posterior distributions of $\phi_{ij}$ and $z_{j}$ are evaluated in the micro E step, while the optimal hyperparameters are evaluated in the micro M step, constituting jointly the macro M step that feeds back into the macro E step.

The aim of this algorithm is to infer the effective structure parameters J and H; this is supported by the optimal ρ that maximize the evidence function lnP(s|ρ). The optimal values of the latent variables also contribute important insight, determining the type of each neuron (excitatory/inhibitory—z) and the existence of links (ϕ taking the value {0,1} for each link). Our approach involves a two-layered EM algorithm for the estimation of hyperparameters and (latent) variables as illustrated in Fig. 7 comprising a “macro” and a “micro” EM algorithms, the latter is being used to perform the M-step of the macro EM algorithm. In the macro E step, the posterior of the effective structure variables, J and H, is evaluated. To make the algorithm tractable we focus on the most likely values, determined using gradient descent and the derivatives $\frac{\partial }{\partial J_{ij}}\ln\;P(s\;|\;\rho )$ and $\frac{\partial }{\partial H_{i}}\ln\;P(s\;|\;\rho )$, given the hyperparameters ρ found in the macro M step. The macro M step maximizes the expected complete-data log-likelihood of the evidence function with respect to the hyperparameters ρ, to determine the optimal values $\rho^{*}$ through the secondary EM process; the micro EM process iterates between the expectation of the probability distributions of the latent variables $\phi_{ij}$ and $z_{j}$, and maximization of the hyperparameters ρ.

Parameters obtained by the macro E step are treated as fixed in the macro M step. In the micro E step, we use the current hyperparameters $\rho_{\mathrm{old}}$ (the notation old refers to values obtained from the maximization step in the micro EM iteration part) and the current effective structure $J^{*}$ and $H^{*}$ to evaluate the posterior probabilities $P_{\mathrm{old}}(\phi_{ij})$ and $P_{\mathrm{old}}(z_{\;j})$ of the latent variables $\phi_{ij}$ and $z_{j}$. Specifically, the probability distributions of the latent variables are given by


(8) $$P_{\mathrm{old}}\left(z_{\;j}\right)\propto p\left({\left\{J_{lj}^{*}\right\}}_{l}\;|\;z_{\;j},\rho \right)p\left(z_{\;j}\;|\;\rho \right),$$

(9) $$P_{\mathrm{old}}\left(\phi_{ij}\right)=\sum_{z_{\;j}}P_{\mathrm{old}}\left(\phi_{ij}\;|\;z_{\;j}\right)P_{\mathrm{old}}\left(z_{\;j}\right).$$

While in the micro M step, one uses $P_{\mathrm{old}}(\phi_{ij})$ and $P_{\mathrm{old}}(z_{\;j})$ evaluated in Eqs. 8 and 9 to determine the optimal hyperparameters that maximize the expected complete-data log-likelihood


(10) $$\begin{array}{rl} Q & =\ln\;P\left(s\;|\;J,H\right)+\ln\;p\left(H\;|\;\vec{\rho }\right)\\ & \;+\sum_{\;j}\sum_{z_{\;j}}\sum_{{\left\{\phi_{ij}\right\}}_{i}}p\left({\left\{\phi_{ij}\right\}}_{i},z_{\;j}\;|\;{\left\{J_{ij}^{*}\right\}}_{i},\rho \right)\ln\;p\left({\left\{J_{ij}^{*}\right\}}_{i},{\left\{\phi_{ij}\right\}}_{i},z_{\;j}\;|\;\rho \right). \end{array}$$

In particular, the hyperparameters $\gamma ,\mu_{J}^{+},v_{J}^{+},\mu_{J}^{-},v_{J}^{-},\mu_{H},v_{H}$ are obtained by setting the corresponding derivatives of Q to zero, while the decay parameter *a* is estimated using gradient descent.

In general, the inference and optimization framework consists of iteratively executing the macro E and M steps until convergence. Within each macro M-step, the hyperparameters are evaluated by iteratively conducting the micro E step and M step until convergence. Finally, one decides on the structure parameters, neuronal type, and link existence by choosing the highest probability states. Notably, for a more sensible starting point, the initial conditions of the hyperparameters ρ can be evaluated using the inferred J and H by using MLE. Additionally, it is worth mentioning that if rapid convergence is preferred over absolute accuracy, the number of macro EM iterations can be limited to one, which effectively results in MAP estimation.

### In silico data generation

Emulated neuronal data have been generated with a spiking neuronal network model previously used to model the network growth and activity of biological neuronal cultures (2, 46). The existing network growth model has been adapted to incorporate the effect of inhomogeneous environments on the network connectivity, thus making the emulated data replicate experimental calcium-recorded results on biological neuronal cultures in inhomogeneous environments (32).

Briefly, network growth is modeled by placing *N* neurons in a nonoverlapping manner on a surface and modeling axon growth from each neuron by concatenating line segments, in which segment *i* is placed with a random angle $\varphi_{i}=\varphi_{i-1}+\sigma_{\varphi }N(0,1)$ with respect to the previous segment i−1. Once an axon segment of neuron *j* is placed within a radius $r_{\mathrm{soma}}\approx 7.5\;\mu m$ of another neuron *i*, a connection $W_{ij}$ is made with a probability *α*. The strength of the connection is drawn from a Gaussian distribution with a mean and standard deviation depending on the neuron type. Inhibitory neurons make up 20% of the network, the remainders are excitatory.

Neuronal dynamics is modeled using the Izhikevich model neuron (37), with added synapse dynamics


(11) $$\frac{dP_{i}}{dt}=-\frac{P_{i}}{\tau_{P}}+\beta R_{i}\delta (v_{i}-v_{th})$$

(12) $$\frac{dR_{i}}{dt}=\frac{1-R_{i}}{\tau_{R}}-\gamma R_{i}\delta (v_{i}-v_{th}).$$

The quantity $\sum_{j}W_{\;ji}P_{i}$ represents the postsynaptic potential induced by the neuron *i*, and $R_{i}$ is the corresponding synaptic neurotransmitter reserve.

### In vitro data generation—experimental methods

#### PDMS topographical reliefs

Topographical patterns were generated by pouring liquid polydimethylsiloxane (PDMS) on specially designed printed circuit board molds shaped as parallel tracks 300μm wide, 70μm high and separated by 200μm (32). PDMS was cured at 100°C for 2 h, separated from the mold, and perforated with sterile punchers to set 4 PDMS cylinders 2 mm in diameter and 0.5 mm high that contained the inverse topographical pattern of the mold. The 4 cylinders were then evenly distributed on a glass coverslip 13 mm in diameter, autoclaved, and coated with PLL. Flat PDMS substrates were also prepared to investigate the impact of topography.

#### Preparation of in vitro neuronal cultures

*Rat primary cultures for patterned networks on PDMS substrates—*Sprague–Dawley rat primary neurons (Charles River Laboratories, France) from embryonic cortices at days 18–19 of development were used in all experiments. Manipulation and dissection of the embryonic cortices were carried out under ethical order B-RP-094/15–7125 of the Animal Experimentation Ethics Committee (CEEA) of the University of Barcelona and in accordance with the regulations of the Generalitat de Catalunya (Spain). Dissection was carried out identically as described in (32). Briefly, cortices were dissected in ice-cold L-15 medium (Gibco), enriched with 0.6% glucose and 0.5% gentamicin (Sigma-Aldrich). Brain cortices were first isolated from the meninges and then mechanically dissociated by repeated pipetting. The resulting dissociated neural progenitors were plated on a set of precoated Poly-L-Lysine (PLL, 10 mg/mL, Sigma-Aldrich) PDMS topographical substrates in the presence of plating medium, which ensured both the development of neurons and glial cells. A density of a half cortex per $1.3\;{\mathrm{cm}}^{2}$ (glass coverslip area) was seeded. This step corresponded to day in vitro (DIV) 0. Two hours after plating, cells were transduced with adeno-associated viruses bearing the genetically encoded calcium fluorescence indicator GCaMP6s under the Synapsin-I promoter, so that only mature neurons expressed the indicator. At DIV 5 the proliferation of glial cells was restricted by incorporating 0.5% FUDR in the culture medium for two more days. From DIV 7 onwards, cells were maintained in a minimum essential medium supplemented with horse serum (Sigma). This medium was changed periodically every 3 days. Cultures were incubated at 37°C, 5% CO_2_, and 95% humidity.

*Mouse primary neuronal cultures for network inference—*13 mm glass coverslips (VWR) were sterilized in 70% Ethanol for 30 min. Coverslips were then transferred to a biological safety cabinet and dried completely before coating for 2 h with 0.02% Poly-L-Ornithine (Sigma). This was washed once with sterile ddH_2_O, and then 20μg/mL murine laminin was added overnight. P0-P2 C57BL/6 mice were used for the network inference study. All animal procedures were approved by Aston University Bioethics Committee and performed in accordance with the United Kingdom Animals Scientific Procedures act of 1986 and current EU legislation. The 3 Rs, replacement, refinement, and reduction were considered for planning all animal procedures. The experiment was carried out at Aston University. Cortices were dissected in ice-cold HBSS (Gibco) containing 1% Penicillin/streptomycin (P/S). Meninges were removed before dissection of both cortices, which were then each cut into eight pieces for enzymatic digestion. Cortices were transferred into 37°C prewarmed HBSS containing 25 U/mL papain (Sigma), 2μg/mL DNAse (Sigma) and L-Cysteine (Sigma). These were incubated at 37°C for 30 min, gently moving and rocking the tube every 7.5 min. Papain solution was removed, and washed twice for 5 min each, at 37°C in MEM (Gibco) + 10% Horse serum (Sigma) + Glutamax (Gibco) + 1% P/S. Cortices were then mechanically dissociated with glass, fire-polished pipettes, to produce homogenous cell suspension. Cells were passed through a 70μm cell strainer (Appleton Woods) to remove any remaining meninges or large clusters of cells or debris. Cells were counted, and seeded onto Poly-L-Ornithine/MuLAM coated glass coverslips at a density of 400,000 cells/cm^2^. After 2 h, media was replaced with Neurobasal (Gibco) + 1% B27 (Gibco) + 1% Glutamax (Gibco) + 1% P/S. Cells were fed every 4 days with a half media change.

#### Intracellular calcium fluorescence imaging

Rat neuronal cultures shaped as ∅ 2 mm PDMS discs allowed the monitoring of the whole network along development. Spontaneous neuronal network activity was recorded using wide-field fluorescence microscopy in combination with the GCaMP6s indicator. Although the networks contained both neurons and glia, only neurons were visualized. Recordings were carried out at DIV 17 for 15 min on a Zeiss Axiovert C25 inverted microscope equipped with a high-speed camera (Hamamatsu Orca Flash 4.0) in combination with an optical zoom. Recordings were carried out at room temperature with the camera software Hokawo 2.10 at 33 frames per second (fps), 8-bit grayscale format, and a size of 1,024×1,024 pixels.

For mouse neuronal cultures, cells were loaded with 10μM Fluo4-AM in DMSO (Invitrogen) for 40 min at 37°C. Coverslips were then transferred onto an upright Nikon FN1 microscope and images were acquired using a Crest Optics XLight V3 spinning disk confocal and a Teledyne Photometrics Kinetix high-speed camera, and in an area of $300\times 300\;\mu m^{2}$. The setup was controlled through Micro-Manager (47). Cultures were perfused with 37°C heated Artificial CSF (aCSF) as a control, and an increase of 2.5 mM KCl to increase baseline activity. Cultures were settled for 5 min before recording commenced at 10 Hz for 10 min. aCSF solution containing the following (in mM): NaCl 120, NaHCO_3_ 25, KCl 2, KH_2_PO_4_ 1.25, MgSO_4_ 1, and CaCl_2_ 2. aCSF chemicals were obtained from Sigma-Aldrich (48).

#### Data analysis

Calcium fluorescence recordings were analyzed with the NETCAL software (49, 50) run in MATLAB in combination with custom-made packages. To analyze the data, and as described in (32), Regions of Interest (ROIs) were first laid on the area covered by each culture. For rat primary cultures, ROIs were shaped as a 20×20 grid centered at the culture and extending its entire 2 mm circular shape, while for mouse primary cultures ROIs corresponded to individual neurons. Next, the average fluorescence trace within each ROI was extracted as a function of time, corrected from drifts, and normalized. Sharp peaks in the fluorescence signals revealed neuronal activations, which were detected using the Schmitt trigger method, finally leading to a binarized time series of neuronal activity in which “1” indicated the presence of neuronal activity and “0” its absence.

## Immunocytochemistry

This technique was used to identify the position of neuronal cell bodies in culture and extract their fluorescence trace with precision. Neuronal cultures were fixed for 20 min with 4% PFA (Sigma) at room temperature. After washing with PBS, the samples were incubated with a blocking solution containing 0.03% Triton (Sigma) and 5% normal donkey serum (Jackson Immunoresearch) in PBS for 45 min at room temperature. To visualize the neuronal nuclei, the samples were incubated with primary antibodies, against the neuronal marker NeuN (M1406, Sigma), diluted in blocking solution, and incubated overnight at 4°C. Cy3-conjugated secondary antibody against rabbit (711-165-152, Jackson Immunoresear) was diluted in blocking solution and incubated for 90 min at room temperature. Then, cultures were rinsed with PBS and mounted using DAPI-fluoromount–G (ShouternBiotech). Immunocytochemical images were acquired on a Zeiss confocal microscope.

## Supplementary Material

pgae565_Supplementary_Data

## Data Availability

All data presented in this paper are available from https://doi.org/10.17036/researchdata.aston.ac.uk.00000635. This includes the Python file containing the derived algorithm, as well as both the in silico and in vitro neuronal firing data being studied.

## Footnotes

a This method is accurate only when coupling strengths are relatively small or when the activation function is smooth.

## Preprints

This manuscript is available as a preprint at: https://arxiv.org/abs/2402.18788.
