# Supplementary material for: Inferring structure of cortical neuronal networks from activity data: A statistical physics approach
Source: PNAS Nexus. 2024 Dec 19;4(1):pgae565. doi: 10.1093/pnasnexus/pgae565 (PMC11713615; doi:10.1093/pnasnexus/pgae565)
Supplement: pgae565_Supplementary_Data [file pgae565_supplementary_data.pdf]

Inferring Structure of Cortical Neuronal Networks from Activity Data: A Statistical Physics Approach

H. F. Po, A. M. Houben, A-C. Haeb, D. R. Jenkins, E. J. Hill, H. R. Parri, J. Soriano, and D. Saad

Ho Fai Po
E-mail: h.po@aston.ac.uk

This PDF file includes:

Supporting text
Figs. S1 to S7
Tables S1 to S2
SI References

Supporting Information Text

1. Generalized maximum likelihood for effective network inference

In this section, we detail the derivation of a variant of the *generalized maximum likelihood* (GML), also known as Type II maximum likelihood or evidence approximation. Consider a neuronal network with N interacting neurons. Denote a discrete variable $s_i^t = \{+1, -1\}$ when neuron i spikes or is silent at a discrete time step t, respectively, for $i = 1, \ldots, N$. The conditional probability of the neuronal activities of all neurons at time t, given the activities of all neurons at the previous time step $t-1$, is as follows:

$$P\left(\boldsymbol{s}^t \left| \boldsymbol{s}^{t-1}, \boldsymbol{J}, \boldsymbol{H}\right.\right) = \prod_{i=1}^{N} P\left(s_i^t \left| \boldsymbol{s}^{t-1}, \boldsymbol{J}, H_i\right.\right) = \prod_{i=1}^{N} \frac{\exp\left[\left(H_i + \sum_j J_{ij} s_j^{t-1}\right) s_i^t\right]}{2\cosh\left(H_i + \sum_j J_{ij} s_j^{t-1}\right)}$$

$$= \exp\left\{\sum_i^N \left[\left(H_i + \sum_j J_{ij} s_j^{t-1}\right) s_i^t - \ln\left[2\cosh\left(H_i + \sum_j J_{ij} s_j^{t-1}\right)\right]\right]\right\}. \quad [1]$$

Here, $\boldsymbol{J} = \{J_{ij}\}$, and each J_{ij} denotes the directed coupling strength from neuron j to neuron i. The coupling strength can be understood as the effective synaptic strength from neuron j to neuron i. Additionally, we denote H_i as the external local field acting on neuron i, where the term $H_i - \sum_j J_{ij}$ can be interpreted as the baseline activity of neuron i when all neighboring neurons of i are silent in the previous time step.

Now, let us consider the mathematical model and latent variables defined in the main text. The variables are as follows: $z_j = \{+1, -1\}$ represents the excitatory/inhibitory type of neuron j, respectively; $\phi_{ij} = 1, 0$ indicates whether the a directed connection from neuron j to neuron i exists or not; $\gamma \in [0, 1]$ represents the fraction of excitatory neurons; θ_{ij} is the prior probability for the existence of a link from neuron j to neuron i; $a \in \mathbb{R}^+$ is a decay parameter; $l_{ij} = l_{ji}$ represents the physical distance between neurons i and j; $\mu_J^{\pm}$ and $v_J^{\pm} \in \mathbb{R}$ are the mean and variance of the distribution of coupling strength $\boldsymbol{J}$ for excitatory and inhibitory neurons, respectively; μ_H and $v_H \in \mathbb{R}$ are the mean and variance of the distribution for $\boldsymbol{H}$. We generally denote prior probabilities by the lower case p, and the log of the prior distributions for all variables can be defined as follows:

$$\ln p\left(z_i \left| \gamma\right.\right) = \delta_{z_i,+1} \ln \gamma + \delta_{z_i,-1} \ln(1-\gamma); \quad [2]$$

$$\ln p\left(\phi_{ij} \left| a\right.\right) = \delta_{\phi_{ij},1}\left(\ln \theta_{ij} - a l_{ij}\right) + \delta_{\phi_{ij},0} \ln\left(1 - \theta_{ij} e^{-a l_{ij}}\right); \quad [3]$$

$$\ln p\left(H_i \left| \mu_H, v_H\right.\right) = \frac{-\left(H_i - \mu_H\right)^2}{2 v_H} - \ln\sqrt{2\pi v_H}; \quad [4]$$

$$\ln p\left(J_{ij} \left| \phi_{ij}, z_j, \mu_J^{\pm}, v_J^{\pm}\right.\right) = \delta_{\phi_{ij},0} \ln \delta\left(J_{ij}\right) + \delta_{\phi_{ij},1} \sum_{\varsigma=\pm 1} \delta_{z_j,\varsigma} \mathbb{1}_{\varsigma J_{ij}>0}\left[\frac{-\left(\ln \zeta J_{ij} - \mu_J^{\zeta}\right)^2}{2 v_J^{\zeta}} - \ln\sqrt{2\pi v_J^{\zeta}}\right] \quad [5]$$

$$\approx \delta_{\phi_{ij},0}\left[\frac{-J_{ij}^2}{2\epsilon} - \ln\sqrt{2\pi\epsilon}\right] + \delta_{\phi_{ij},1} \sum_{\varsigma=\pm 1} \delta_{z_j,\varsigma} \mathbb{1}_{\varsigma J_{ij}>0}\left[\frac{-\left(\ln \zeta J_{ij} - \mu_J^{\zeta}\right)^2}{2 v_J^{\zeta}} - \ln\sqrt{2\pi v_J^{\zeta}}\right]. \quad [6]$$

The coefficient θ_{ij} describes how likely i and j are connected due to physical constraints (e.g. existence of patterned substrates), which is set to be 1 for the homogeneous environment; while ϵ is a small real number to approximate $\delta\left(J_{ij}\right)$ by a standard normal distribution with small variance, such that existing connections admit the log-normal distributions and absent connections are close to zero. As illustrated in Fig. S1, the prior distribution $p\left(J_{ij}\right)$ follows a mixture of three distributions: two log-normal distributions with positive and negative means to represent the excitatory and inhibitory connections, respectively; and a standard normal distribution with small variance to represent the absent connections. Log-normal distributions are selected for effective connections to fit the biological understanding and act as a constraint to restrict J_{ij} can only be positive (negative) when $z_j = 1$ ($z_j = -1$). For simplicity, we denote $\boldsymbol{\rho} = \left\{\gamma, a, \mu_J^+, v_J^+, \mu_J^-, v_J^-, \mu_H, v_H\right\}$ as the set of all introduced hyperparameters, where the $\pm$ signs refers to the groups representing excitatory and inhibitory neurons. We remark that a and $\{\theta_{ij}\}$ will be fixed for the whole process with values that agree with biological understanding, which we set to $a = 0.1$ in all of our cases. We will optimize the hyperparameters except a and $\{\theta_{ij}\}$ using GML as discussed below.

First, the evidence function, or the marginalized log-likelihood, is given by

$$\ln P\left(\boldsymbol{s} \left| \boldsymbol{\rho}\right.\right) = \ln \int d\boldsymbol{J}\, d\boldsymbol{H}\, P\left(\boldsymbol{s}, \boldsymbol{J}, \boldsymbol{H} \left| \boldsymbol{\rho}\right.\right). \quad [7]$$

Our objective is to maximize the log-likelihood function but do it in stages; we first consider the evidence function of data given the hyperparameters. To achieve this, we apply an Expectation Maximization (EM)-inspired algorithm and call this the macro EM. The expected complete log-likelihood with respect to $\boldsymbol{\rho}$ is defined as

$$\int d\boldsymbol{J}\, d\boldsymbol{H}\, P\left(\boldsymbol{J}, \boldsymbol{H} \left| \boldsymbol{s}, \boldsymbol{\rho}^{\Xi}\right.\right) \ln\left[P\left(\boldsymbol{s}, \boldsymbol{J}, \boldsymbol{H} \left| \boldsymbol{\rho}\right.\right)\right] \quad [8]$$

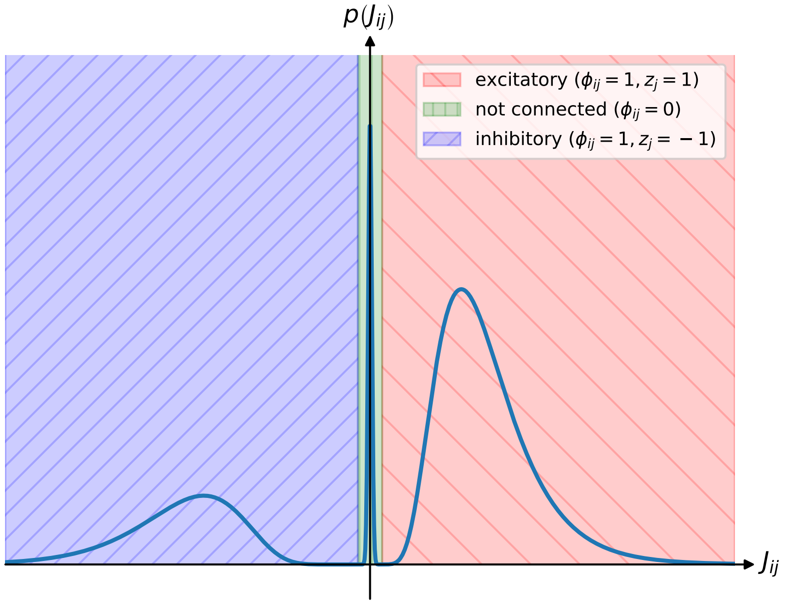

Fig. S1. A sketch illustrating the shape of the prior distribution $p\left(J_{ij}\right)$ for the directed coupling strength. $P\left(J_{ij}\right)$ follows a mixture of three distributions: a log-normal distribution with positive mean to represent the excitatory connections ($\phi_{ij} = 1,\, z_j = 1$), as shown in the red shaded region; a log-normal distribution with negative mean to represent the inhibitory connections ($\phi_{ij} = 1,\, z_j = -1$), as shown in the blue shaded region; a Gaussian distribution with zero mean and small variance, approximately a Dirac delta function, to represent absent connections ($\phi_{ij} = 0$), as shown in the green shaded region.

In general, we aim to find the maximum log-evidence probability and the posterior probability $P\left(\boldsymbol{J}, \boldsymbol{H} \,\middle|\, \boldsymbol{s}, \boldsymbol{\rho}^{\Xi}\right)$ in the macro E-step (originally the expectation probability) by fixing $\boldsymbol{\rho}^{\Xi}$ obtained in the previous macro M-step; and we then evaluate and update $\boldsymbol{\rho}^{\Xi}$ as well as the posterior probabilities of the latent variables, $\boldsymbol{\phi}$ and $\boldsymbol{z}$, in the macro M-step.

A. Macro E-step. In the macro E-step, we evaluate the posterior probability $P\left(\boldsymbol{J}, \boldsymbol{H} \,\middle|\, \boldsymbol{s}, \boldsymbol{\rho}^{\Xi}\right)$, given the the fixed $\boldsymbol{\rho}^{\Xi}$ obtained in the previous macro M-step. Since it is computationally challenging to obtain the full distribution of $P\left(\boldsymbol{J}, \boldsymbol{H} \,\middle|\, \boldsymbol{s}, \boldsymbol{\rho}^{\Xi}\right)$, we approximate using the saddle point method by its most likely values,

$$\boldsymbol{J}^{*}, \boldsymbol{H}^{*} = \underset{\{\boldsymbol{J},\boldsymbol{H}\}}{\operatorname{argmax}} P\left(\boldsymbol{J}, \boldsymbol{H} \,\middle|\, \boldsymbol{s}, \boldsymbol{\rho}^{\Xi}\right) = \underset{\{\boldsymbol{J},\boldsymbol{H}\}}{\operatorname{argmax}} \ln P\left(\boldsymbol{J}, \boldsymbol{H} \,\middle|\, \boldsymbol{s}, \boldsymbol{\rho}^{\Xi}\right), \qquad [9]$$

relying on the large system size, which gives the maximum posterior probability.

Then, we approximate $P\left(\boldsymbol{J}, \boldsymbol{H} \,|\, \boldsymbol{s}, \boldsymbol{\rho}\right) \approx \delta\left(\boldsymbol{J} - \boldsymbol{J}^{*}\right) \delta\left(\boldsymbol{H} - \boldsymbol{H}^{*}\right)$ after $\boldsymbol{J}^{*}$ and $\boldsymbol{H}^{*}$ are found. Finding the maximum of $P\left(\boldsymbol{J}, \boldsymbol{H} \,|\, \boldsymbol{s}, \boldsymbol{\rho}\right)$ is equivalent to finding the maximum for $\ln P\left(\boldsymbol{J}, \boldsymbol{H} \,|\, \boldsymbol{s}, \boldsymbol{\rho}\right) = \ln \sum_{\boldsymbol{\phi}} \sum_{\boldsymbol{z}} P\left(\boldsymbol{J}, \boldsymbol{H}, \boldsymbol{\phi}, \boldsymbol{z} \,\middle|\, \boldsymbol{s}, \boldsymbol{\rho}^{\Xi}\right)$ which is intractable. Instead, we find the optimal $\boldsymbol{J}^{*}$ and $\boldsymbol{H}^{*}$ that maximize the expected complete log likelihood (in the notation, we omit the dependence on $\boldsymbol{s}$ and $\boldsymbol{\rho}^{\Xi}$):

$$\mathcal{Q}_{\Xi}\left(\boldsymbol{J}, \boldsymbol{H}\right) = \sum_{\boldsymbol{\phi}} \sum_{\boldsymbol{z}} P_{\Xi}\left(\boldsymbol{\phi}, \boldsymbol{z}\right) \ln P\left(\boldsymbol{J}, \boldsymbol{H}, \boldsymbol{\phi}, \boldsymbol{z} \,\middle|\, \boldsymbol{s}, \boldsymbol{\rho}^{\Xi}\right), \qquad [10]$$

where $P_{\Xi}\left(\cdot\right)$ denotes the posterior probability $P\left(\cdot \,\middle|\, \boldsymbol{s}, \boldsymbol{J}, \boldsymbol{H}, \boldsymbol{\rho}^{\Xi}\right)$ evaluated in the previous macro M-step, for brevity.

Then, simplifying Eq. [10], we get

$$\begin{aligned}
\mathcal{Q}_{\Xi}(\boldsymbol{J},\boldsymbol{H}) &= \sum_{\boldsymbol{\phi}}\sum_{\boldsymbol{z}} P_{\Xi}(\boldsymbol{\phi},\boldsymbol{z}) \ln\left[P\left(\boldsymbol{s}\left|\boldsymbol{J},\boldsymbol{H},\boldsymbol{\phi},\boldsymbol{z},\boldsymbol{\rho}^{\Xi}\right.\right) p\left(\boldsymbol{J},\boldsymbol{H},\boldsymbol{\phi},\boldsymbol{z}\left|\boldsymbol{\rho}^{\Xi}\right.\right)/P\left(\boldsymbol{s}\left|\boldsymbol{\phi},\boldsymbol{z},\boldsymbol{\rho}^{\Xi}\right.\right)\right] \\
&= \sum_{\boldsymbol{\phi}}\sum_{\boldsymbol{z}} P_{\Xi}(\boldsymbol{\phi},\boldsymbol{z}) \ln\left[P\left(\boldsymbol{s}\left|\boldsymbol{J},\boldsymbol{H}\right.\right) p\left(\boldsymbol{J},\boldsymbol{H}\left|\boldsymbol{\phi},\boldsymbol{z},\boldsymbol{\rho}^{\Xi}\right.\right) p\left(\boldsymbol{\phi},\boldsymbol{z}\left|\boldsymbol{\rho}^{\Xi}\right.\right)/P\left(\boldsymbol{s}\left|\boldsymbol{\phi},\boldsymbol{z},\boldsymbol{\rho}^{\Xi}\right.\right)\right] \\
&= \ln P\left(\boldsymbol{s}\left|\boldsymbol{J},\boldsymbol{H}\right.\right) + \sum_{\boldsymbol{\phi}}\sum_{\boldsymbol{z}} P_{\Xi}(\boldsymbol{\phi},\boldsymbol{z}) \ln\prod_{ij} p\left(J_{ij}\left|\phi_{ij},z_j,\boldsymbol{\rho}^{\Xi}\right.\right) \\
&\quad + \sum_{\boldsymbol{\phi}}\sum_{\boldsymbol{z}} P_{\Xi}(\boldsymbol{\phi},\boldsymbol{z}) \ln\prod_{i} p\left(H_i\left|\boldsymbol{\rho}^{\Xi}\right.\right) + \sum_{\boldsymbol{\phi}}\sum_{\boldsymbol{z}} P_{\Xi}(\boldsymbol{\phi},\boldsymbol{z}) \ln\prod_{ij} p\left(\phi_{ij}\left|\boldsymbol{\rho}^{\Xi}\right.\right) \\
&\quad + \sum_{\boldsymbol{\phi}}\sum_{\boldsymbol{z}} P_{\Xi}(\boldsymbol{\phi},\boldsymbol{z}) \ln\prod_{i} p\left(z_i\left|\boldsymbol{\rho}^{\Xi}\right.\right) - \sum_{\boldsymbol{\phi}}\sum_{\boldsymbol{z}} P_{\Xi}(\boldsymbol{\phi},\boldsymbol{z}) \ln P\left(\boldsymbol{s}\left|\boldsymbol{\phi},\boldsymbol{z},\boldsymbol{\rho}^{\Xi}\right.\right) \\
&= \ln P\left(\boldsymbol{s}\left|\boldsymbol{J},\boldsymbol{H}\right.\right) + \sum_{ij}\sum_{z_j=\pm1}\sum_{\phi_{ij}=1,0} P_{\Xi}\left(\phi_{ij}\left|z_j\right.\right) P_{\Xi}(z_j) \ln p\left(J_{ij}\left|\phi_{ij},z_j,\boldsymbol{\rho}^{\Xi}\right.\right) \\
&\quad + \sum_{ij}\sum_{z_j=\pm1}\sum_{\phi_{ij}=1,0} P_{\Xi}\left(\phi_{ij}\left|z_j\right.\right) P_{\Xi}(z_j) \ln p\left(\phi_{ij}\left|\boldsymbol{\rho}^{\Xi}\right.\right) \\
&\quad + \sum_{j}\sum_{z_j=\pm1} P_{\Xi}(z_j) \ln\prod_{i} p\left(z_i\left|\boldsymbol{\rho}^{\Xi}\right.\right) + \sum_{i}\ln p\left(H_i\left|\boldsymbol{\rho}^{\Xi}\right.\right) - \sum_{\boldsymbol{\phi}}\sum_{\boldsymbol{z}} P_{\Xi}(\boldsymbol{\phi},\boldsymbol{z}) \ln P\left(\boldsymbol{s}\left|\boldsymbol{\phi},\boldsymbol{z},\boldsymbol{\rho}^{\Xi}\right.\right) \\
&= \sum_{t}\sum_{i}\left\{\left(H_i + \sum_{j} J_{ij}s_j^{t-1}\right)s_i^t - \ln\left[2\cosh\left(H_i + \sum_{j} J_{ij}s_j^{t-1}\right)\right]\right\} \\
&\quad + \sum_{i}\sum_{j}\sum_{z_j=\pm1}\sum_{\phi_{ij}=1,0} P_{\Xi}\left(\phi_{ij}\left|z_j\right.\right) P_{\Xi}(z_j) \left\{\delta_{\phi_{ij},0}\left[-\left(J_{ij}\right)^2/(2\epsilon) - \ln\sqrt{2\pi\epsilon}\right]\right. \\
&\qquad \left. + \delta_{\phi_{ij},1}\sum_{\varsigma=\pm1}\delta_{z_j,\varsigma}\mathbb{1}_{\varsigma J_{ij}>0}\left[-\left(\ln\zeta J_{ij} - \mu_J^{\zeta,\Xi}\right)^2/\left(2v_J^{\zeta}\right) - \ln\sqrt{2\pi v_J^{\zeta}}\right]\right\} \\
&\quad + \sum_{i}\sum_{j}\sum_{z_j=\pm1}\sum_{\phi_{ij}=1,0} P_{\Xi}\left(\phi_{ij}\left|z_j\right.\right) P_{\Xi}(z_j) \left[\delta_{\phi_{ij},1}\left(\ln\theta_{ij} - al_{ij}\right) + \delta_{\phi_{ij},0}\ln\left(1-\theta_{ij}e^{-al_{ij}}\right)\right] \\
&\quad + \sum_{i}\sum_{z_i=\pm1} P_{\Xi}(z_j)\left[\delta_{z_i,+1}\ln\gamma + \delta_{z_i,-1}\ln(1-\gamma)\right] \\
&\quad + \sum_{i}\left\{\frac{-\left(H_i-\mu_H\right)^2}{2v_H^{\Xi}} - \ln\sqrt{2\pi v_H^{\Xi}}\right\} - \sum_{\boldsymbol{\phi}}\sum_{\boldsymbol{z}} P_{\Xi}(\boldsymbol{\phi},\boldsymbol{z}) \ln P\left(\boldsymbol{s}\left|\boldsymbol{\phi},\boldsymbol{z},\boldsymbol{\rho}^{\Xi}\right.\right).
\end{aligned} \qquad [11]$$

The optimization process is carried out through gradient descent, in particular,

$$(\delta\boldsymbol{J},\delta\boldsymbol{H}) = \eta\nabla\mathcal{Q}_{\Xi}(\boldsymbol{J},\boldsymbol{H}), \qquad [12]$$

where $\nabla\mathcal{Q}_{\Xi}(\boldsymbol{J},\boldsymbol{H}) = \left(\left\{\frac{\partial\mathcal{Q}_{\Xi}}{\partial J_{ij}}\right\}_{ij}, \left\{\frac{\partial\mathcal{Q}_{\Xi}}{\partial H_i}\right\}_{i}\right)$ and η is the decaying learning rate; the gradients are given as

$$\begin{aligned}
\frac{\partial\mathcal{Q}_{\Xi}}{\partial J_{ij}} &= T\left[\left\langle s_i^t s_j^{t-1}\right\rangle_t - \left\langle s_j^{t-1}\tanh\left(H_i + \sum_{k} J_{ik}s_k^{t-1}\right)\right\rangle_t\right] \\
&\quad - \sum_{z_j=\pm1}\sum_{\phi_{ij}=1,0} P_{\Xi}\left(\phi_{ij}\left|z_j\right.\right) P_{\Xi}(z_j)\left\{\delta_{\phi_{ij},0}\frac{J_{ij}}{\epsilon} + \delta_{\phi_{ij},1}\sum_{\varsigma=\pm1}\delta_{z_j,\varsigma}\mathbb{1}_{\varsigma J_{ij}>0}\frac{\ln\zeta J_{ij} - \mu_J^{\zeta,\Xi}}{J_{ij}v_J^{\zeta}}\right\},
\end{aligned} \qquad [13]$$

and

$$\frac{\partial\mathcal{Q}_{\Xi}}{\partial H_i} = T\left[\left\langle s_i^t\right\rangle_t - \left\langle\tanh\left(H_i + \sum_{j} J_{ij}s_j^{t-1}\right)\right\rangle_t\right] - \frac{H_i - \mu_H}{v_H^{\Xi}}. \qquad [14]$$

Upon convergence, the optimal $\boldsymbol{J}^*$ and $\boldsymbol{H}^*$ is fixed for the evaluation of $\boldsymbol{\rho}$ and the posterior probabilities in the following macro M-step.

B. Macro M-step. Fixing the peak values $\boldsymbol{J}^*$ and $\boldsymbol{H}^*$ obtained in the previous macro E-step, the log-likelihood $\boldsymbol{\rho}$ becomes

$$\begin{aligned}\int d\boldsymbol{J}\, d\boldsymbol{H}\; P\left(\boldsymbol{J},\boldsymbol{H}\,\middle|\,\boldsymbol{s},\boldsymbol{\rho}^{\Xi}\right)\ln\left[P\left(\boldsymbol{s},\boldsymbol{J},\boldsymbol{H}\,|\boldsymbol{\rho}\right)\right] &= \int d\boldsymbol{J}\, d\boldsymbol{H}\;\delta\left(\boldsymbol{J}-\boldsymbol{J}^*\right)\delta\left(\boldsymbol{H}-\boldsymbol{H}^*\right)\ln\left[P\left(\boldsymbol{s},\boldsymbol{J},\boldsymbol{H}\,|\boldsymbol{\rho}\right)\right]\\ &= \ln\left[P\left(\boldsymbol{s},\boldsymbol{J}^*,\boldsymbol{H}^*\,|\boldsymbol{\rho}\right)\right]\\ &= \ln\left[\sum_{\boldsymbol{\phi}}\sum_{\boldsymbol{z}}P\left(\boldsymbol{s},\boldsymbol{J}^*,\boldsymbol{H}^*,\boldsymbol{\phi},\boldsymbol{z}\,|\boldsymbol{\rho}\right)\right]. \end{aligned}\tag{15}$$

The objective of the macro M-step is to find the optimal value $\boldsymbol{\rho}^*$ that maximizes Eq. [15]. Interpreting $\boldsymbol{\phi}$ and $\boldsymbol{z}$ as latent variables, we apply a second EM algorithm, termed the micro EM, to obtain $\boldsymbol{\rho}^*$. The expected complete data log-likelihood is then given by

$$\begin{aligned}\mathcal{Q} &= \sum_{\boldsymbol{\phi}}\sum_{\boldsymbol{z}}P\left(\boldsymbol{\phi},\boldsymbol{z}\,\middle|\,\boldsymbol{s},\boldsymbol{J}^*,\boldsymbol{H}^*,\boldsymbol{\rho}^{\text{old}}\right)\ln P\left(\boldsymbol{s},\boldsymbol{J}^*,\boldsymbol{H}^*,\boldsymbol{\phi},\boldsymbol{z}\,|\boldsymbol{\rho}\right)\\ &= \sum_{\boldsymbol{\phi}}\sum_{\boldsymbol{z}}P\left(\boldsymbol{\phi},\boldsymbol{z}\,\middle|\,\boldsymbol{s},\boldsymbol{J}^*,\boldsymbol{H}^*,\boldsymbol{\rho}^{\text{old}}\right)\ln\left[P\left(\boldsymbol{s}\,|\boldsymbol{J}^*,\boldsymbol{H}^*,\boldsymbol{\phi},\boldsymbol{z},\boldsymbol{\rho}\right)p\left(\boldsymbol{J}^*,\boldsymbol{H}^*,\boldsymbol{\phi},\boldsymbol{z}\,|\boldsymbol{\rho}\right)\right]\\ &= \sum_{\boldsymbol{\phi}}\sum_{\boldsymbol{z}}P\left(\boldsymbol{\phi},\boldsymbol{z}\,\middle|\,\boldsymbol{s},\boldsymbol{J}^*,\boldsymbol{H}^*,\boldsymbol{\rho}^{\text{old}}\right)\ln\left[P\left(\boldsymbol{s}\,|\boldsymbol{J}^*,\boldsymbol{H}^*\right)p\left(\boldsymbol{H}^*\,|\boldsymbol{\rho}\right)p\left(\boldsymbol{J}^*,\boldsymbol{\phi},\boldsymbol{z}\,|\boldsymbol{\rho}\right)\right]\\ &= \ln P\left(\boldsymbol{s}\,|\boldsymbol{J}^*,\boldsymbol{H}^*\right)+\ln p\left(\boldsymbol{H}^*\,|\boldsymbol{\rho}\right)+\sum_{\boldsymbol{\phi}}\sum_{\boldsymbol{z}}P\left(\boldsymbol{\phi},\boldsymbol{z}\,\middle|\,\boldsymbol{s},\boldsymbol{J}^*,\boldsymbol{H}^*,\boldsymbol{\rho}^{\text{old}}\right)\ln p\left(\boldsymbol{J}^*,\boldsymbol{\phi},\boldsymbol{z}\,|\boldsymbol{\rho}\right),\end{aligned}\tag{16}$$

where $\boldsymbol{\rho}^{\text{old}}$ is the values of the hyperparameters evaluated in the previous micro M-step. For simplicity, we denote $P\left(\cdot\,\middle|\,\boldsymbol{s},\boldsymbol{J}^*,\boldsymbol{H}^*,\boldsymbol{\rho}^{\text{old}}\right)$ as $P_{\text{old}}\left(\cdot\right)$. Consider the term

$$\begin{aligned}\sum_{\boldsymbol{\phi}}\sum_{\boldsymbol{z}}P_{\text{old}}\left(\boldsymbol{\phi},\boldsymbol{z}\right)\ln p\left(\boldsymbol{J}^*,\boldsymbol{\phi},\boldsymbol{z}\,|\boldsymbol{\rho}\right) &= \sum_{\boldsymbol{\phi}}\sum_{\boldsymbol{z}}P_{\text{old}}\left(\boldsymbol{\phi},\boldsymbol{z}\right)\ln\left[p\left(\boldsymbol{J}^*\,|\boldsymbol{\phi},\boldsymbol{z},\boldsymbol{\rho}\right)p\left(\boldsymbol{\phi},\boldsymbol{z}\,|\boldsymbol{\rho}\right)\right]\\ &= \sum_{\boldsymbol{\phi}}\sum_{\boldsymbol{z}}P_{\text{old}}\left(\boldsymbol{\phi}\,|\boldsymbol{z}\right)P_{\text{old}}\left(\boldsymbol{z}\right)\ln p\left(\boldsymbol{J}^*\,|\boldsymbol{\phi},\boldsymbol{z},\boldsymbol{\rho}\right)\\ &\quad+\sum_{\boldsymbol{\phi}}\sum_{\boldsymbol{z}}P_{\text{old}}\left(\boldsymbol{\phi}\,|\boldsymbol{z}\right)P_{\text{old}}\left(\boldsymbol{z}\right)\ln p\left(\boldsymbol{\phi}\,|\boldsymbol{z},\boldsymbol{\rho}\right)+\sum_{\boldsymbol{z}}P_{\text{old}}\left(\boldsymbol{z}\right)\ln p\left(\boldsymbol{z}\,|\boldsymbol{\rho}\right).\end{aligned}\tag{17}$$

By considering the terms in Eq. [17] one by one:

$$\sum_{\{\phi\}}\sum_{\{z\}} P_{\text{old}}(\boldsymbol{\phi}|\boldsymbol{z}) P_{\text{old}}(\boldsymbol{z}) \ln p(\boldsymbol{J}^*|\boldsymbol{\phi},\boldsymbol{z},\boldsymbol{\rho}) = \sum_{\{\phi\}}\sum_{\{z\}} P_{\text{old}}(\boldsymbol{\phi}|\boldsymbol{z}) P_{\text{old}}(\boldsymbol{z}) \ln \prod_j p\left(\left\{J^*_{kj}\right\}_k \left|\{\phi_{kj}\}_k, z_j, \boldsymbol{\rho}\right.\right)$$

$$= \sum_j \left[\sum_{\{\phi\}}\sum_{\{z\}} P_{\text{old}}(\boldsymbol{\phi}|\boldsymbol{z}) P_{\text{old}}(\boldsymbol{z}) \ln p\left(\left\{J^*_{kj}\right\}_k \left|\{\phi_{kj}\}_k, z_j, \boldsymbol{\rho}\right.\right)\right]$$

$$= \sum_j \sum_{\{\phi\}}\sum_{\{z\}} \left\{\prod_u \left[P_{\text{old}}\left(\{\phi_{ku}\}_k|z_u\right) P_{\text{old}}(z_u)\right] \ln p\left(\left\{J^*_{kj}\right\}_k \left|\{\phi_{kj}\}_k, z_j, \boldsymbol{\rho}\right.\right)\right\}$$

$$= \sum_j \sum_{\{\phi\}}\sum_{\{z\}} \left\{\left[\prod_{u\neq j} P_{\text{old}}\left(\{\phi_{ku}\}_k|z_u\right) P_{\text{old}}(z_u)\right] P_{\text{old}}\left(\{\phi_{kj}\}_k|z_j\right) P_{\text{old}}(z_j) \ln p\left(\left\{J^*_{kj}\right\}_k \left|\{\phi_{kj}\}_k, z_j, \boldsymbol{\rho}\right.\right)\right\}$$

$$= \sum_j \sum_{\left\{\phi_{kj}\right\}_k} \sum_{z_j=\pm1} P_{\text{old}}\left(\{\phi_{kj}\}_k|z_j\right) P_{\text{old}}(z_j) \ln p\left(\left\{J^*_{kj}\right\}_k \left|\{\phi_{kj}\}_k, z_j, \boldsymbol{\rho}\right.\right)$$

$$= \sum_j \sum_{z_j=\pm1} P_{\text{old}}(z_j) \sum_{\left\{\phi_{kj}\right\}_k} P_{\text{old}}\left(\{\phi_{kj}\}_k|z_j\right) \ln \prod_i p\left(J^*_{ij}|\phi_{ij}, z_j, \boldsymbol{\rho}\right)$$

$$= \sum_j \sum_i \sum_{z_j=\pm1} P_{\text{old}}(z_j) \sum_{\left\{\phi_{kj}\right\}_k} \prod_v P_{\text{old}}(\phi_{vj}|z_j) \ln p\left(J^*_{ij}|\phi_{ij}, z_j, \boldsymbol{\rho}\right)$$

$$= \sum_j \sum_i \sum_{z_j=\pm1} P_{\text{old}}(z_j) \sum_{\left\{\phi_{kj}\right\}_k} \left[\prod_{v\neq i} P_{\text{old}}(\phi_{vj}|z_j)\right] P_{\text{old}}(\phi_{ij}|z_j) \ln p\left(J^*_{ij}|\phi_{ij}, z_j, \boldsymbol{\rho}\right)$$

$$= \sum_j \sum_i \sum_{z_j=\pm1} P_{\text{old}}(z_j) \sum_{\phi_{ij}=1,0} P_{\text{old}}(\phi_{ij}|z_j) \ln p\left(J^*_{ij}|\phi_{ij}, z_j, \boldsymbol{\rho}\right)$$

$$= \sum_i \sum_j \sum_{z_j=\pm1} \sum_{\phi_{ij}=1,0} P_{\text{old}}(\phi_{ij}|z_j) P_{\text{old}}(z_j) \ln p\left(J^*_{ij}|\phi_{ij}, z_j, \boldsymbol{\rho}\right)$$

$$= \sum_i \sum_j \sum_{z_j=\pm1} \sum_{\phi_{ij}=1,0} P_{\text{old}}(\phi_{ij}|z_j) P_{\text{old}}(z_j) \left\{\delta_{\phi_{ij},0}\left[-\left(J^*_{ij}\right)^2/(2\epsilon) - \ln\sqrt{2\pi\epsilon}\right]\right.$$

$$\left. + \delta_{\phi_{ij},1} \sum_{\varsigma=\pm1} \delta_{z_j,\varsigma} \mathbb{1}_{\varsigma J^*_{ij}>0}\left[-\left(\ln\zeta J^*_{ij} - \mu^\zeta_J\right)^2/\left(2v^\zeta_J\right) - \ln\sqrt{2\pi v^\zeta_J}\right]\right\},$$

and

$$\sum_{\phi}\sum_{z} P_{\text{old}}(\boldsymbol{\phi}|\boldsymbol{z}) P_{\text{old}}(\boldsymbol{z}) \ln p(\boldsymbol{\phi}|\boldsymbol{z},\boldsymbol{\rho}) = \sum_j \sum_{\phi}\sum_{z} P_{\text{old}}(\boldsymbol{\phi}|\boldsymbol{z}) P_{\text{old}}(\boldsymbol{z}) \ln p\left(\{\phi_{kj}\}_k|z_j,\boldsymbol{\rho}\right)$$

$$= \sum_j \sum_{\phi}\sum_{z} \left\{\prod_{u\neq j}\left[P_{\text{old}}\left(\{\phi_{ku}\}_k|z_u\right) P_{\text{old}}(z_u)\right]\right\} P_{\text{old}}\left(\{\phi_{kj}\}_k|z_j\right) P_{\text{old}}(z_j) \ln p\left(\{\phi_{kj}\}_k|z_j,\boldsymbol{\rho}\right)$$

$$= \sum_j \sum_{\left\{\phi_{kj}\right\}_k} \sum_{z_j=\pm1} P_{\text{old}}\left(\{\phi_{kj}\}_k|z_j\right) P_{\text{old}}(z_j) \ln p\left(\{\phi_{kj}\}_k|z_j,\boldsymbol{\rho}\right)$$

$$= \sum_j \sum_i \sum_{z_j=\pm1} P_{\text{old}}(z_j) \sum_{\left\{\phi_{kj}\right\}_k} \left[\prod_{v\neq i} P_{\text{old}}(\phi_{vj}|z_j)\right] P_{\text{old}}(\phi_{ij}|z_j) \ln p(\phi_{ij}|z_j,\boldsymbol{\rho})$$

$$= \sum_j \sum_i \sum_{z_j=\pm1} P_{\text{old}}(z_j) \sum_{\phi_{ij}=1,0} P_{\text{old}}(\phi_{ij}|z_j) \ln p(\phi_{ij}|z_j,\boldsymbol{\rho})$$

$$= \sum_i \sum_j \sum_{z_j=\pm1} \sum_{\phi_{ij}=1,0} P_{\text{old}}(\phi_{ij}|z_j) P_{\text{old}}(z_j) \ln p(\phi_{ij}|z_j,\boldsymbol{\rho})$$

$$= \sum_i \sum_j \sum_{z_j=\pm1} \sum_{\phi_{ij}=1,0} P_{\text{old}}(\phi_{ij}|z_j) P_{\text{old}}(z_j) \left[\delta_{\phi_{ij},1}\left(\ln\theta_{ij} - al_{ij}\right) + \delta_{\phi_{ij},0}\ln\left(1-\theta_{ij}e^{-al_{ij}}\right)\right],$$

and

$$
\begin{aligned}
&\sum_{\boldsymbol{z}} P_{\text{old}}\left(\boldsymbol{z}\right) \ln p\left(\boldsymbol{z}\left|\boldsymbol{\rho}\right.\right) \\
&= \sum_i \sum_{\boldsymbol{z}} \left[\prod_{u\neq i} P_{\text{old}}\left(z_u\right)\right] P_{\text{old}}\left(z_i\right) \ln p\left(z_i\left|\boldsymbol{\rho}\right.\right) \\
&= \sum_i \sum_{z_i=\pm 1} P_{\text{old}}\left(z_i\right) \ln p\left(z_i\left|\boldsymbol{\rho}\right.\right) \\
&= \sum_i \sum_{z_i=\pm 1} P_{\text{old}}\left(z_i\right) \left[\delta_{z_i,+1}\ln\gamma + \delta_{z_i,-1}\ln\left(1-\gamma\right)\right].
\end{aligned}
$$

We remark that the posterior probability of ϕ_{ij} depends on z_j due to the dependence of ϕ_{ij} on J_{ij}, which depends on z_j. Therefore, the expected complete data log-likelihood simplifies to

$$
\begin{aligned}
\mathcal{Q} &= \ln P\left(\boldsymbol{s}\left|\boldsymbol{J}^*,\boldsymbol{H}^*\right.\right) + \ln p\left(\boldsymbol{H}^*\left|\boldsymbol{\rho}\right.\right) + \sum_{\boldsymbol{\phi}}\sum_{\boldsymbol{z}} P\left(\boldsymbol{\phi},\boldsymbol{z}\left|\boldsymbol{s},\boldsymbol{J}^*,\boldsymbol{H}^*,\boldsymbol{\rho}^{\text{old}}\right.\right) \ln p\left(\boldsymbol{J}^*,\boldsymbol{\phi},\boldsymbol{z}\left|\boldsymbol{\rho}\right.\right) \\
&= \sum_t \sum_i \left\{\left(H_i^* + \sum_j J_{ij}^* s_j^{t-1}\right) s_i^t - \ln\left[2\cosh\left(H_i^* + \sum_j J_{ij}^* s_j^{t-1}\right)\right]\right\} \\
&\quad + \sum_i \left\{\frac{-\left(H_i^*-\mu_H\right)^2}{2v_H} - \ln\sqrt{2\pi v_H}\right\} \\
&\quad + \sum_i \sum_j \sum_{z_j=\pm 1} \sum_{\phi_{ij}=1,0} P_{\text{old}}\left(\phi_{ij}\left|z_j\right.\right) P_{\text{old}}\left(z_j\right) \left\{\delta_{\phi_{ij},0}\left[-\left(J_{ij}^*\right)^2/\left(2\epsilon\right) - \ln\sqrt{2\pi\epsilon}\right]\right. \\
&\qquad \left. + \delta_{\phi_{ij},1} \sum_{\varsigma=\pm 1} \delta_{z_j,\varsigma} \mathbb{1}_{\varsigma J_{ij}^*>0} \left[-\left(\ln\zeta J_{ij}^* - \mu_J^\zeta\right)^2/\left(2v_J^\zeta\right) - \ln\sqrt{2\pi v_J^\zeta}\right]\right\} \\
&\quad + \sum_i \sum_j \sum_{z_j=\pm 1} \sum_{\phi_{ij}=1,0} P_{\text{old}}\left(\phi_{ij}\left|z_j\right.\right) P_{\text{old}}\left(z_j\right) \left[\delta_{\phi_{ij},1}\left(\ln\theta_{ij} - al_{ij}\right) + \delta_{\phi_{ij},0}\ln\left(1-\theta_{ij}e^{-al_{ij}}\right)\right] \\
&\quad + \sum_i \sum_{z_i=\pm 1} P_{\text{old}}\left(z_i\right)\left[\delta_{z_i,+1}\ln\gamma + \delta_{z_i,-1}\ln\left(1-\gamma\right)\right],
\end{aligned} \tag{18}
$$

allowing us to perform the micro EM more efficiently.

B.1. Micro E-step. In the micro E-step, we evaluate the posterior probabilities $P\left(\boldsymbol{\phi}\left|\boldsymbol{z},\boldsymbol{s},\boldsymbol{J}^*,\boldsymbol{H}^*,\boldsymbol{\rho}^{\text{old}}\right.\right)$ and $P\left(\boldsymbol{z}\left|\boldsymbol{s},\boldsymbol{J}^*,\boldsymbol{H}^*,\boldsymbol{\rho}^{\text{old}}\right.\right)$ for $\boldsymbol{\phi}$ and $\boldsymbol{z}$, conditioned on $\boldsymbol{\rho}^{\text{old}}$ evaluated in the previous micro M-step as well as $\boldsymbol{J}^*$ and $\boldsymbol{H}^*$ obtained in the last macro E-step. In particular,

$$
P\left(\boldsymbol{\phi}\left|\boldsymbol{z},\boldsymbol{s},\boldsymbol{J}^*,\boldsymbol{H}^*,\boldsymbol{\rho}^{\text{old}}\right.\right) = \prod_{ij} P\left(\phi_{ij}\left|\boldsymbol{z},\boldsymbol{s},\boldsymbol{J}^*,\boldsymbol{H}^*,\boldsymbol{\rho}^{\text{old}}\right.\right), \text{ where} \tag{19}
$$

$$
\begin{aligned}
P\left(\phi_{ij}\left|\boldsymbol{z},\boldsymbol{s},\boldsymbol{J}^*,\boldsymbol{H}^*,\boldsymbol{\rho}^{\text{old}}\right.\right) &= p\left(\phi_{ij}\left|z_j,J_{ij}^*,\boldsymbol{\rho}^{\text{old}}\right.\right) \\
&= \frac{p\left(J_{ij}^*\left|\phi_{ij},z_j,\boldsymbol{\rho}^{\text{old}}\right.\right) p\left(\phi_{ij}\left|z_j,\boldsymbol{\rho}^{\text{old}}\right.\right)}{\sum_{\phi'_{ij}} p\left(J_{ij}^*\left|\phi'_{ij},z_j,\boldsymbol{\rho}^{\text{old}}\right.\right) p\left(\phi'_{ij}\left|z_j,\boldsymbol{\rho}^{\text{old}}\right.\right)};
\end{aligned} \tag{20}
$$

and

$$
P\left(\boldsymbol{z}\left|\boldsymbol{s},\boldsymbol{J}^*,\boldsymbol{H}^*,\boldsymbol{\rho}^{\text{old}}\right.\right) = \prod_j P\left(z_j\left|\boldsymbol{s},\boldsymbol{J}^*,\boldsymbol{H}^*,\boldsymbol{\rho}^{\text{old}}\right.\right), \text{ where} \tag{21}
$$

$$
\begin{aligned}
P\left(z_j\left|\boldsymbol{s},\boldsymbol{J}^*,\boldsymbol{H}^*,\boldsymbol{\rho}^{\text{old}}\right.\right) &= p\left(z_j\left|\left\{J_{ij}^*\right\}_i,\boldsymbol{\rho}^{\text{old}}\right.\right) \\
&= \frac{p\left(\left\{J_{ij}^*\right\}_i\left|z_j,\boldsymbol{\rho}^{\text{old}}\right.\right) p\left(z_j\left|\boldsymbol{\rho}^{\text{old}}\right.\right)}{\sum_{z'_j}\left[p\left(\left\{J_{ij}^*\right\}_i\left|z'_j,\boldsymbol{\rho}^{\text{old}}\right.\right) p\left(z'_j\left|\boldsymbol{\rho}^{\text{old}}\right.\right)\right]} \\
&= \frac{p\left(z_j\left|\boldsymbol{\rho}^{\text{old}}\right.\right)\prod_i\left[\sum_{\phi'_{ij}=1,0} p\left(J_{ij}^*\left|\phi'_{ij},z_j,\boldsymbol{\rho}^{\text{old}}\right.\right) p\left(\phi'_{ij}\left|z_j,\boldsymbol{\rho}^{\text{old}}\right.\right)\right]}{\sum_{z'_j} p\left(z'_j\left|\boldsymbol{\rho}^{\text{old}}\right.\right)\prod_i\left[\sum_{\phi'_{ij}=1,0} p\left(J_{ij}^*\left|\phi'_{ij},z'_j,\boldsymbol{\rho}^{\text{old}}\right.\right) p\left(\phi'_{ij}\left|z'_j,\boldsymbol{\rho}^{\text{old}}\right.\right)\right]}.
\end{aligned} \tag{22}
$$

The updated posterior probabilities are then passed to the micro M-step for evaluating the updated $\boldsymbol{\rho}$.

B.2. Micro M-step. In the micro M-step, we use the posterior probabilities $P\left(\boldsymbol{\phi}\left|\boldsymbol{z}, \boldsymbol{s}, \boldsymbol{J}^*, \boldsymbol{H}^*, \boldsymbol{\rho}^{\text{old}}\right.\right)$ and $P\left(\boldsymbol{z}\left|\boldsymbol{s}, \boldsymbol{J}^*, \boldsymbol{H}^*, \boldsymbol{\rho}^{\text{old}}\right.\right)$, as well as the $\boldsymbol{J}^*$ and $\boldsymbol{H}^*$ obtained in the last macro E-step, to update the values of $\boldsymbol{\rho}$. The derivatives of $\mathcal{Q}$ with respect to all variables in $\boldsymbol{\rho}$ except a give:

$$\frac{\partial \mathcal{Q}}{\partial \gamma} = \sum_i \sum_{z_i=\pm 1} P_{\text{old}}\left(z_i\right)\left[\delta_{z_i,+1}\frac{1}{\gamma} + \delta_{z_i,-1}\frac{1}{1-\gamma}\right], \tag{23}$$

$$\frac{\partial \mathcal{Q}}{\partial \mu_J^\zeta} = \sum_i \sum_j P_{\text{old}}\left(\phi_{ij}=1\left|z_j=\zeta\right.\right) P_{\text{old}}\left(z_j=\zeta\right) \mathbb{1}_{\zeta J_{ij}^*>0} \frac{\ln \zeta J_{ij}^* - \mu_J^\zeta}{v_J^\zeta}, \text{ for } \zeta = \pm 1 \tag{24}$$

$$\frac{\partial \mathcal{Q}}{\partial v_J^\zeta} = \sum_i \sum_j P_{\text{old}}\left(\phi_{ij}=1\left|z_j=\zeta\right.\right) P_{\text{old}}\left(z_j=\zeta\right) \mathbb{1}_{\zeta J_{ij}^*>0} \frac{\left(\ln \zeta J_{ij}^* - \mu_J^\zeta\right)^2 - v_j^\zeta}{2\left(v_J^\zeta\right)^2}, \text{ for } \zeta = \pm 1 \tag{25}$$

$$\frac{\partial \mathcal{Q}}{\partial \mu_H} = \sum_i \left[\frac{H_i^* - \mu_H}{v_H}\right], \tag{26}$$

$$\frac{\partial \mathcal{Q}}{\partial \mu_H} = \sum_i \left[\frac{\left(H_i^* - \mu_H\right)^2 - v_H}{2 v_H^2}\right]. \tag{27}$$

By setting them to zero, we obtain

$$\gamma = \frac{\sum_i P_{\text{old}}\left(z_i=+1\right)}{N} \tag{28}$$

$$\mu_J^\zeta = \frac{\sum_i \sum_j P_{\text{old}}\left(\phi_{ij}=1\left|z_j=\zeta\right.\right) P_{\text{old}}\left(z_j=\zeta\right) \mathbb{1}_{\zeta J_{ij}^*>0} \ln \zeta J_{ij}^*}{\sum_i \sum_j P_{\text{old}}\left(\phi_{ij}=1\left|z_j=\zeta\right.\right) P_{\text{old}}\left(z_j=\zeta\right) \mathbb{1}_{\zeta J_{ij}^*>0}}, \text{ for } \zeta = \pm 1 \tag{29}$$

$$v_J^\zeta = \frac{\sum_i \sum_j P_{\text{old}}\left(\phi_{ij}=1\left|z_j=\zeta\right.\right) P_{\text{old}}\left(z_j=\zeta\right) \mathbb{1}_{\zeta J_{ij}^*>0} \left(\ln \zeta J_{ij}^* - \mu_J^\zeta\right)^2}{\sum_i \sum_j P_{\text{old}}\left(\phi_{ij}=1\left|z_j=\zeta\right.\right) P_{\text{old}}\left(z_j=\zeta\right) \mathbb{1}_{\zeta J_{ij}^*>0}}, \text{ for } \zeta = \pm 1 \tag{30}$$

$$\mu_H = \frac{\sum_i H_i^*}{N}, \tag{31}$$

$$v_H = \frac{\sum_i \left(H_i^* - \mu_H\right)^2}{N}. \tag{32}$$

The micro E and M-steps are iterating alternatively until convergence, and the converged hyperparameters $\boldsymbol{\rho}^\Xi$, as well as the posterior probabilities $P\left(\boldsymbol{\phi}\left|\boldsymbol{z}, \boldsymbol{s}, \boldsymbol{J}^*, \boldsymbol{H}^*, \boldsymbol{\rho}^\Xi\right.\right)$ and $P\left(\boldsymbol{z}\left|\boldsymbol{s}, \boldsymbol{J}^*, \boldsymbol{H}^*, \boldsymbol{\rho}^\Xi\right.\right)$ are then fixed to find the optimal $\boldsymbol{J}^*$ and $\boldsymbol{H}^*$ in the next macro E-step.

As an overview – we execute the macro E and M steps iteratively until convergence, and within each macro M-step, we evaluate the hyperparameters and the posterior probabilities by conducting the micro E-step and M-step iteratively until convergence. Afterward, the structure parameters for neuronal type $\boldsymbol{z}^*$ and link existence $\boldsymbol{\phi}^*$ are selected by choosing the highest probability states from the micro E-step, i.e.

$$z_j^* = \underset{z_j'}{\text{argmax}} P\left(z_j'\left|\boldsymbol{s}, \boldsymbol{J}^*, \boldsymbol{H}^*, \boldsymbol{\rho}^\Xi\right.\right), \text{ and} \tag{33}$$

$$\begin{aligned}\phi_{ij}^* &= \underset{\phi_{ij}'}{\text{argmax}} P\left(\phi_{ij}'\left|\boldsymbol{s}, \boldsymbol{J}^*, \boldsymbol{H}^*, \boldsymbol{\rho}^\Xi\right.\right) \\ &= \underset{\phi_{ij}'}{\text{argmax}} \sum_{z_j'} P\left(\phi_{ij}'\left|z_j', \boldsymbol{s}, \boldsymbol{J}^*, \boldsymbol{H}^*, \boldsymbol{\rho}^\Xi\right.\right) P\left(z_j'\left|\boldsymbol{s}, \boldsymbol{J}^*, \boldsymbol{H}^*, \boldsymbol{\rho}^\Xi\right.\right).\end{aligned} \tag{34}$$

To provide clarity and detail on how the variables used in our model are defined, assigned, and evaluated, we have included Table S1 to summarize all relevant variables. The table outlines each variable's type, description, and corresponding value, offering an organized overview of the parameters, hyperparameters, and latent variables that are essential for inferring neuronal dynamics and connectivity.

2. Alternative inference algorithms for the kinetic Ising model

Extensive studies have been carried out and many algorithms have been derived for the structure inference problem of the kinetic Ising model, such as various mean-field approaches (1–3), the message passing algorithm (4) and maximum likelihood estimation (MLE). Nevertheless, most of them are less suitable for real neuronal network inference. In this section, we will first introduce these methods briefly and explain why they are inadequate for neuronal network inference. Next, we will present results of structural inference for synthetic data under different conditions.

Variable	Type	Description	Value
s_i^t	Data	Activity of neuron i at time t	$+1$ (spike), -1 (silent)
J_{ij}	Parameter	Directed coupling strength from neuron j to i	Real value - target of the inference algorithm
H_i	Parameter	External local field acting on neuron i	Real value - target of the inference algorithm
z_j	Latent variable	Excitatory/inhibitory type of neuron j	Real value - target of the inference algorithm
ϕ_{ij}	Latent variable	Effective connectivity between neurons i and j	Real value - target of the inference algorithm
a	Hyperparameter	Decay parameter for distant neurons' connectivity likelihood	Fixed at 0.1
θ_{ij}	Hyperparameter	Prior probability of link existence	Homogeneous: fixed at 0.9; Stripes: 0.5^{m_d}, where m_d is the number of stripes separating neurons i and j
γ	Hyperparameter	Fraction of excitatory neurons	Fixed at 0.8
$\mu_J^{\pm}$	Hyperparameter	Mean of the prior distribution of $\boldsymbol{J}$ for excitatory/inhibitory connections	evaluated by Eq. [29] using results from MLE for MAP; follows macro EM procedure for GML
$\nu_J^{\pm}$	Hyperparameter	Variance of the prior distribution of $\boldsymbol{J}$ for excitatory/inhibitory connections	evaluated by Eq. [30] using results from MLE for MAP; follows macro EM procedure for GML
μ_H	Hyperparameter	Mean of the prior distribution of $\boldsymbol{H}$	evaluated by Eq. [31] using results from MLE for MAP; follows macro EM procedure for GML
ν_H	Hyperparameter	Variance of the prior distribution of $\boldsymbol{H}$	evaluated by Eq. [32] using results from MLE for MAP; follows macro EM procedure for GML

Table S1. Summary of variables used in the model, including their types, descriptions, corresponding values and how hey are obtained. The table provides an overview of the parameters, hyperparameters and latent variables relevant for inferring neuronal dynamics and connectivity.

Mean-field approaches, including naïve mean-field (nMF) (1, 2), the Thouless-Anderson-Palmer (TAP) approach (1), and the exact mean-field (exactMF) (3), aim to estimate the inferred couplings $\boldsymbol{J}$ and external fields $\boldsymbol{H}$ by approximating the intractable true distribution in Eq. (1) by a tractable one. Both nMF and TAP rely on weak-coupling expansion, meaning that J_{ij} are small in magnitude, following a Gaussian distribution with mean zero and small variance. While nMF follows the conventional mean-field equation for the ordinary Ising model where all s_i are at equilibrium, TAP improves the accuracy of estimation by nMF through considering the Onsager reaction term (5, 6), solving an extra set of cubic equations in addition to the nMF equation, whose roots are not guaranteed to exist when J_{ij} are not weak. On the other hand, exactMF provides a better approximation of the couplings by establishing an exact mean-field equation using the central limit theorem and solving a set of integral equations iteratively, whose roots are also not guaranteed to exist. Furthermore, exactMF requires that the equal-time correlations $\left\langle s_i^t s_j^t \right\rangle_t - \left\langle s_i^t \right\rangle_t \left\langle s_j^t \right\rangle_t$ for all i, j and the quantity $\sum_j J_{ij} J_{ji}$ to be of order $1/\sqrt{N}$ for all i. Although mean-field approaches can provide decent approximations in the weak-couplings case, they cannot provide good quality inferred coupling values when these essential conditions do not hold, which is typically the case for real neuronal networks. In our model we assume effective synaptic strengths that follow a mixture of three probability distributions, corresponding to excitatory, inhibitory, and non-effective connections, as shown in Fig. S1. The existence of distinct excitatory and inhibitory neurons and links does not allow for couplings emanating from a single neuron to be mixed, adding a severe constraint on the couplings to be inferred. Thus, mean-field approaches are not optimally suited for neuronal network inference.

Maximum likelihood estimation (MLE) aims to find the optimal values of $\boldsymbol{J}$ and $\boldsymbol{H}$ such that the likelihood function $P\left(\boldsymbol{s}^t \middle| \boldsymbol{s}^{t-1}, \boldsymbol{J}, \boldsymbol{H}\right)$ defined in Eq. (1) is maximized. It is worthwhile mentioning that since MLE optimizes the probability $P\left(\boldsymbol{s}^t \middle| \boldsymbol{s}^{t-1}, \boldsymbol{J}, \boldsymbol{H}\right)$ without any constraints on the parameters $\boldsymbol{J}$ and $\boldsymbol{H}$, the resulting inferred structure $\boldsymbol{J}$ and $\boldsymbol{H}$ should best explain the observed data. Nevertheless, MLE does not: (1) include the fact that the inferred network is not fully connected (i.e., most $J_{ij} = 0$); (2) restrict $\{J_{ij}\}_j$ to be all non-negative (non-positive) if j is excitatory (inhibitory); (3) impose a particular structure like the stripes-patterned substrate case we show in the main text.

Another promising approach for network inference is the dynamical cavity method (4), which can be understood as an extension of the belief propagation approximation algorithm. This method can provide an accurate approximation to the underlying network even when the couplings J_{ij} are large. However, it only works when the structures are known (connections between neurons are already given), and the connectivity of each neuron is small ($\sum_i \phi_{ij} \ll N$). These criteria are unrealistic for neuronal networks and hence this method is not applicable to our problem.

On the other hand, we emphasize that our proposed methods, MAP and GML, accommodate all of the problems mentioned above. In particular, our methods: (1) work well even when J_{ij} are large; (2) can infer network topology through the directed connectivity ϕ_{ij}; (3) can infer the excitatory and inhibitory type of neurons; (4) take the structure imposed on the culture and network connection generation properties into account, in the form of priors; (5) do not require a *known* sparsely connected structure, but infer the structure from data. Noting that MAP obtains the hyperparameters $\boldsymbol{\rho}$ using Eq. (28 - 32) with structural variables $\boldsymbol{J}$ and $\boldsymbol{H}$ from MLE and executes the Macro EM steps only once, it performs the inference much faster than GML, in general. On the other hand, GML performs the complete EM steps until convergence, in which both hyperparameters $\boldsymbol{\rho}$, and structural variables $\boldsymbol{J}$ and $\boldsymbol{H}$ are optimized; this provides better results, given the constraints and prior information. It is worthwhile mentioning that, as both MAP and GML acquire the values of the hyperparameters automatically, there is no need to assign specific values to $\boldsymbol{\rho}$, except for the connectivity generation control parameter a and the variances $\nu_J^{\pm}$, ν_H. The parameter a always increases through the Macro EM iterations; similarly, variance $\nu_J^{\pm}$ and ν_H always decrease. This is due to the fact that a acts as a control to regulate the magnitude of J_{ij}, while the variances $\nu_J^{\pm}$ and ν_H influence the smoothness of

the solution. As the system progresses through iterations, it seeks to minimize J_{ij} while maximizing the objective function, which causes a to increase and the variances to shrink in order to converge toward a sparser solution. As a result, most J_{ij} will converge to 0 eventually. Therefore, we should fix a to some value and set a lower bound to $\nu_J^{\pm}$ and ν_H. In our case, we fix $a = 0.1$ and $\nu_J^{\pm} = \nu_H = 0.1$. We emphasize that the effect of the prior distributions diminishes as the number of samples increases. Therefore, the fixed value of a and the lower bound for the variances are just arbitrary numbers to make sure the probability $P\left(\boldsymbol{J}, \boldsymbol{H}, \boldsymbol{\phi}, \boldsymbol{z} \,\middle|\, \boldsymbol{s}, \boldsymbol{\rho}^{\Xi}\right)$ does not vanish. Moreover, one can *fix* some of the known variables, such as the ratio γ between inhibitory and excitatory neurons for faster convergence.

To provide a clearer picture of the performance of different algorithms, we generated synthetic data directly from the kinetic Ising model for multiple realizations. In particular, to simulate a cortical network with a suitable ratio between excitatory and inhibitory neurons, we assigned 80% (20%) of the neurons to be excitatory (inhibitory), where $J_{ij} \geq 0$ (≤ 0) for all i if j is an excitatory (inhibitory) neuron. Additionally, the probability of two spins being connected decreases exponentially with the distance between them. The mean values for non-zero J_{ij} for excitatory and inhibitory connections are the corresponding mean μ and -1.4μ respectively, where realizations are generated over different values of μ from 0.11 to 0.71. Data of each realization are generated using Monte-Carlo simulation. We then infer the structures given the synthetic data, using the mean-field methods, MLE, and our MAP and GML approaches, followed by prediction of the structure variables (connectivity, neuronal type, and synaptic strengths) using the variables inferred. To compare the performance, except for the coupling strengths $\boldsymbol{J}$, we also study two macroscopic observables, namely the equal-time covariance matrix $\boldsymbol{C} = \left\{\left\langle s_i^t s_j^t \right\rangle_t\right\}_{i,j}$ and the delayed-time covariance matrix $\boldsymbol{D} = \left\{\left\langle s_i^t s_j^{t-1} \right\rangle_t\right\}_{i,j}$, by evaluating the root mean square error (RMSE) of results obtained by the different inference methods from the ground truth.

In Fig. S2A, B, and C, we show the RMSE of $\boldsymbol{J}$, $\boldsymbol{C}$, and $\boldsymbol{D}$ using different methods, against the scaled quantity $\sqrt{N}\left\langle \sum_j J_{ij}J_{ji} \right\rangle_i$, respectively. We plot the errors against $\sqrt{N}\left\langle \sum_j J_{ij}J_{ji} \right\rangle_i$ because most mean-field approaches are assumed to work only when this quantity is smaller than one, thus this comparison provides a clear criterion for evaluating the performance of these methods. As shown in Fig. S2A, we can see that the RMSE of the inferred coupling strengths increases significantly as $\sqrt{N}\left\langle \sum_j J_{ij}J_{ji} \right\rangle_i$ increases, especially when it is larger than one. Notably, we can see that the RMSE of the inferred $\boldsymbol{J}$ using exactMF increased by more than 10^4 times when $\sqrt{N}\left\langle \sum_j J_{ij}J_{ji} \right\rangle_i$ is larger than one, suggesting that the inferred coupling strengths are extremely inaccurate. This result agrees with the fact that these mean-field approaches are accurate only when $\sqrt{N}\left\langle \sum_j J_{ij}J_{ji} \right\rangle_i$ is smaller than one, which is unrealistic in neuronal networks. On the other hand, we can see that the RMSEs of the inferred $\boldsymbol{J}$ using MAP and GML, are always lower than the other methods, and the result from GML is better than MAP. This suggests that our algorithms perform best for structural inference, while GML performs better than MAP, justifying the reason to perform the macro EM steps instead of a single iteration.

As shown in Fig. S2B and C, similar to the inferred coupling strengths, mean-field approaches start to fail when $\sqrt{N}\left\langle \sum_j J_{ij}J_{ji} \right\rangle_i$ is greater than 1 (the scaling of $\sqrt{N}$ is used since most methods assume weak couplings, such that this scaling make the quantities evaluated to be of $O(1)$), with the RMSE of both $\boldsymbol{C}$ and $\boldsymbol{D}$ increase with $\sqrt{N}\left\langle \sum_j J_{ij}J_{ji} \right\rangle_i$, with MLE explaining the observed data best for all values, for both $\boldsymbol{C}$ and $\boldsymbol{D}$. The reason for that is that MLE is free from all of the constraints imposed to represent a realistic neuronal network, such as the separation of excitatory and inhibitory neurons, the sparsity of connections etc. When $\sqrt{N}\left\langle \sum_j J_{ij}J_{ji} \right\rangle_i$ is smaller than one, both mean-field approaches and MLE perform slightly better than our MAP and GML approaches. When $\sqrt{N}\left\langle \sum_j J_{ij}J_{ji} \right\rangle_i$ exceeds one, MAP and GML outperform mean-field approaches but remain inferior to MLE. While MLE shows a good fit in terms of the macroscopic measures, we want to emphasize that the aim of the study is inferring the effective structure of the sample, including accurate effective synaptic strengths, neuronal type, and effective connectivity that agree with biological understandings. The methods we have devised, particularly MAP and GLM, are much more suitable for this task.

3. Alternative methods for neuronal network inference

In addition to the comparison with Generalized Transfer Entropy (GTE) in the main text, we consider two other commonly used methods for neuronal network inference: the Pairwise Maximum Entropy Model (MEM) and Generalized Linear Models (GLM). While these methods are valuable tools for studying neural activity, their objectives and underlying assumptions differ from those of our approach, making direct comparisons less relevant.

The Pairwise Maximum Entropy Model (MEM) assumes that neuronal firing patterns follow an Ising model, where the goal is to maximize entropy while matching the average firing rates and equal-time correlations observed in the data (7). Despite the surface similarity to our kinetic Ising model, MEM has several key limitations. First, it only considers equal-time interactions between neurons, with symmetric effective coupling strengths (i.e., $J_{ij} = J_{ji}$), meaning MEM cannot describe directed interactions or the temporal influence of one neuron's spike on another's at a subsequent time step, a feature critical to real neuronal dynamics and captured by our methods. Second, MEM only accounts for pairwise interactions and has a higher computational cost, as it requires calculating the full distribution $P(s)$, which scales as 2^N for a network of N neurons. These factors make MEM less suited for inferring realistic neuronal network structures, and it lacks the ability to infer effective

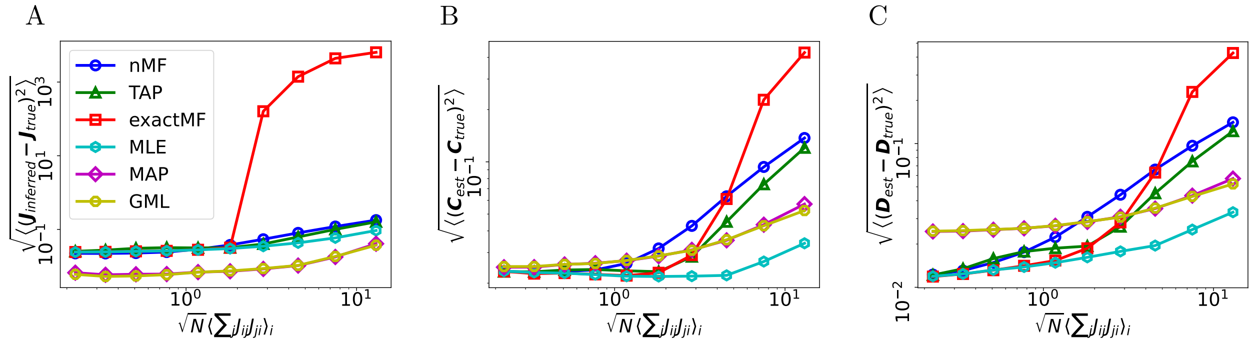

Fig. S2. The average root mean square error (RMSE) of inferred quantities. A: J_{ij}, B: C_{ij}, and C: D_{ij} against $\sqrt{N}\left\langle \sum_j J_{ij}J_{ji} \right\rangle_i$ for synthetic kinetic Ising models. Synthetic data are generated from 10 realizations of kinetic Ising model dynamics with $N = 50$, an excitatory-to-inhibitory ratio of 4:1. The mean values of excitatory and inhibitory non-zero connections are μ and -1.4μ respectively, where μ varies from 0.11 to 0.71. the fraction of effective non-zero links is 6%, and each model was run for 2000 time steps.

synaptic connections and neuronal types, as our methods do.

The Generalized Linear Model (GLM), on the other hand, models firing rates as a function of other neurons' spiking activity at the previous time step (8), making it somewhat analogous to the Maximum Likelihood Estimation (MLE) used in our kinetic Ising model. However, GLM does not infer effective connectivity or neuronal types and focuses solely on firing rates, rather than the precise firing times of individual neurons, which is a core aspect of our approach. Additionally, the synaptic strengths inferred by GLM have a different interpretation than those in our model, as GLM captures average firing rate dependencies rather than microscopic interactions between neurons. For these reasons, we believe that a direct comparison with GLM is not appropriate for our specific objectives of inferring both network structure and neuron types.

Given these fundamental differences, we conclude that while MEM and GLM are valuable in their respective contexts, they are not suitable for extracting the detailed neuronal dynamics and structural inferences that our methods provide. Our approach not only identifies effective connections but also infers neuronal type, offering a more comprehensive framework for understanding neuronal networks.

4. Additional Results

To validate the performance of our method on benchmark data, we generated additional data sets from synthetic and *in silico* models. In particular, to show our inference method performs well on biological neuronal systems compared to the existing methods, we test our approach with synthetic data.

A. Kinetic Ising model. To examine the efficacy of our inference algorithm and compare its performance to that of existing approaches, we first use synthetic data generated directly from a kinetic Ising model (3), a similar model to the celebrated Ising model but where interactions between spins are non-symmetric. In particular, we generate data from a kinetic Ising model with system size $N = 50$. To simulate biological systems, we assigned 80% (20%) of the neurons (or spins) to be excitatory (inhibitory), where $J_{ij} \geq 0$ (≤ 0) for all i if j is an excitatory (inhibitory) neuron. Additionally, the probability of two spins being connected decreases exponentially with the distance between them. Synthetic data is generated using Monte-Carlo simulation for 3000 steps. We then infer the structure variables $\boldsymbol{J}$ given the synthetically-generated Monte-Carlo data. Subsequently, we predict the structure variables (connectivity, neuronal type and synaptic strengths) using the inferred structure and the known underlying variable values. The inference method also requires one to evaluate the equal-time covariance matrix $\boldsymbol{C} = \left\{ \left\langle s_i^t s_j^t \right\rangle_t \right\}_{i,j}$ and the delayed time covariance matrix $\boldsymbol{D} = \left\{ \left\langle s_i^t s_j^{t-1} \right\rangle_t \right\}_{i,j}$; we also use these matrices, of both inferred and true data, to measure the accuracy of the inference method.

We compare our GML approach against the naïve mean field (nMF), maximum likelihood estimation (MLE) and maximum a posteriori (MAP) approach, by plotting the inferred values of J_{ij} against the true value in Fig. S3. Briefly, nMF refers to the simple factorization of the joint probability of all relevant variables into individual probabilities, MLE refers to maximizing the probability of the data given model parameters, MAP to maximizing the probability of parameter values given data and the GML to an iterative process whereby model hyper-parameters and variable values are estimated and optimized recursively.

Fig. S3 shows the accuracy of the inferred synaptic strengths $\boldsymbol{J}$, predicted equal-time covariance matrix $\boldsymbol{C}$ and delayed time covariance matrix $\boldsymbol{D}$; the better the scatter plot aligns with the dotted line $y = x$, the more accurate is the structure parameters' inference and its alignment with the data. We observe that nMF results are inferior in performance compared to the other methods; naïve mean-field works best when J_{ij} are Gaussian distributed with zero mean and small variance, while in the more realistic case, we simulate J_{ij} to follow a mixture of distributions that are far from zero. The predicted values using MLE already align very well with the true values, but many disconnected neuron pairs $J_{ij} = 0$ are inferred to have nonzero values, due to the fact that link existence is not considered separately in MLE.

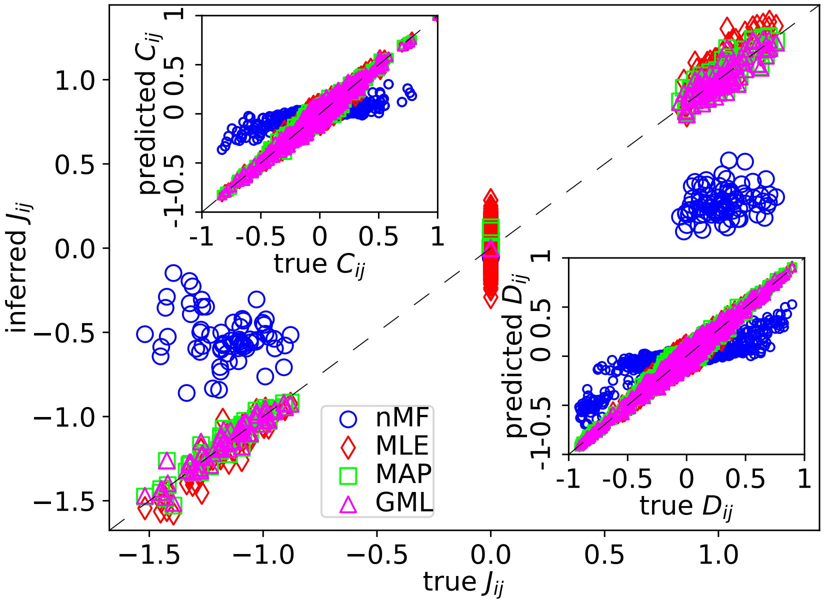

Fig. S3. Inferred coupling strengths $\boldsymbol{J}$ obtained using nMF, MLE, MAP and GML compared to the true coupling strengths generated by a synthetic model. Top left inset: the predicted equal time covariance matrix values $\boldsymbol{C}$ against the true values. Bottom right inset: the predicted delayed time covariance matrix values $\boldsymbol{D}$ against the true values. Synthetic data are generated from a realization of kinetic Ising model simulation with N=50, the ratio between positive and negative $\boldsymbol{J}$ is 1:4, the mean values of positive and negative $\boldsymbol{J}$ are 1 and -1.35 respectively, the fraction of effective links is 6%, and the time steps of the simulation is 3000.

To address the issue of non-existing links we employ the EM algorithm. Remarkably, even after a single macro EM step (jointly with MAP), except for two true zero links, all true zero links are inferred as zero (disconnected). Moreover, if we employ the full GML procedure until convergence, we can see that neuron type classification (excitatory/inhibitory/zero) for all J_{ij} is completely correct. This suggests that our algorithm excels at identifying effective linkages between neurons. In addition, we can see that the error of inference using the GML is significantly lower than those achieved by other methods. The predicted $\boldsymbol{C}$ and $\boldsymbol{D}$ covariance matrices are plotted against the true values as shown in the insets of Fig. S3. The prediction accuracy for $\boldsymbol{C}$ and $\boldsymbol{D}$ are similar for MLE, MAP and GML. These results suggest that our model and inference algorithm are appropriate for structural inference on biological neural networks, especially when J_{ij} follows a mixture of distributions.

B. Experimental data - *In silico* homogeneous network. In the main text, we studied the performance of our algorithm on emulated *in silico* data over patterned substrates and showed that our method performs very well on neuronal type classification and link existence. Noted that patterned substrates restrict neurons from different modules connecting to each other, thus might reduce the solution space of $\boldsymbol{J}$, $\boldsymbol{H}$, $\boldsymbol{z}$ and $\boldsymbol{\phi}$, making the inference problem easier to solve, comparing to homogeneous setup. Therefore, one would expect better inference results to be obtained from the activity data generated from the patterned substrates model. Here we test our algorithm on emulated *in silico* data with homogeneous network structure, to verify the performance of accuracy of neuronal type classification and link existence on non-structured substrates.

A homogeneous neuronal network with 156 neurons is generated using the same emulator as the *in silico* model with patterning substrates studied in the main text. Next, spontaneous neuronal activity data is simulated and used as the input for the effective structure inference using MLE, MAP, and the GML. For the prior distributions, based on the statistical information already known from the *in silico* model, we fix $\gamma = 0.8$, $a = 0.1$ and $\theta_{ij} = 0.9$ for all neurons i and j. The values of the other hyperparameters, which are used for both MAP inference and the initial values of GML, are evaluated using Eq. [29-32], given the $\boldsymbol{J}$ and $\boldsymbol{H}$ inferred by MLE.

Measure	TPR/TNR	PPV/NPV	Random
**Excitatory**	0.81	**0.94**	0.8
**Inhibitory**	0.81	**0.52**	0.2
**Overall**	n/a	**0.81**	0.68

Table S2. Success measures in identifying neuron type: true positive rate (TPR - sensitivity), True negative rate (TNR - specificity), and positive predictive value (PPV) of our *in silico* homogeneous model study.

The connectivity matrix showing the inferred coupling strengths $\boldsymbol{J}$ using GML is shown in Fig. S4C, where inhibitory and excitatory connections are colored in blue and red respectively. Similar to the other results we obtained *in silico* and *in*

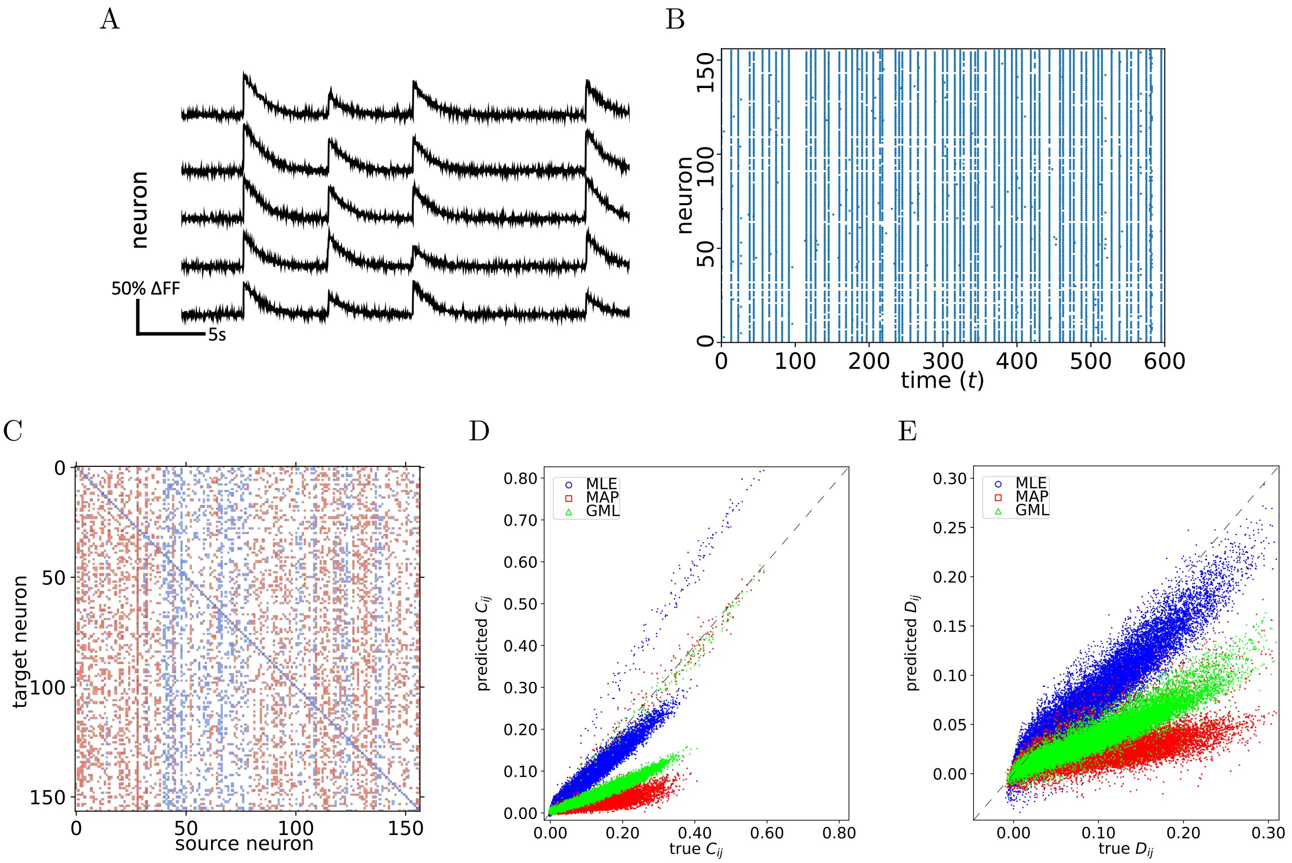

Fig. S4. A: Example *in silico* traces of five neurons in the *in silico* homogeneous model. B: The raster plot displays neuronal activities inferred from the *in silico* traces C: The inferred structure $\boldsymbol{J}$ of the *in silico* homogeneous model obtained using GML and represented by a connectivity matrix. Each entry corresponds to the coupling strength J_{ij}. A positive (negative) strength colored in red (blue) refers to excitatory (inhibitory) signals sent from source (i) to target (j) neurons. D and E: The predicted equal-time covariance $\boldsymbol{C}$ and delayed time covariance $\boldsymbol{D}$ against the true values evaluated from data, respectively.

vitro experiments, we can see that the diagonal connections are mostly inhibitory, which may reflect the fact that neurons are less likely to have consecutive firing due to the reduced potential. To measure the precision of neuronal type prediction, the positive predictive value (PPV), negative predictive value (NPV) of $P\left(z_{\text{true}} \middle| z_{\text{inferred}}\right)$, as well as the true positive rate (TPR) and the true negative rate (TNR) showing $P\left(z_{\text{inferred}} = +/- \,|z_{\text{true}} = +/-\right)$, are shown in Table S2, where excitatory and inhibitory type is the positive case and negative case respectively. The overall predictability is the weighted sum of the probabilities in both cases. We can see that the neuronal type prediction of our method outperforms the prior-based random guess approach, while the predictability of the case of the patterned substrate is slightly higher only. The high predictability suggests that our method still performs very well even in the homogeneous case.

Next, we test the performance of the link existence inference using MAP and GML, and compare them with the results obtained by generalized transfer entropy (GTE) (9). The complete receiver operating characteristic (ROC) curve showing the TPR and FPR of identifying non-zero links using GTE with all threshold values is shown in Fig. S5. The corresponding TPR and FPR using GML are 78% and 21%, respectively, and 60% and 4.7% for MAP, respectively, are indicated by red and green circles in the figure as well. Similar to the main text, our method offers a higher TPR than GTE at the same FPR level, or a lower FPR at the same level of TPR. This suggests that our method performs well in identifying effective links even without the help of the spatial constraints in connectivity.

Similar to what we have done in the main text, we employ the inferred structure $\boldsymbol{J}$ and $\boldsymbol{H}$ using MLE, MAP and GML, to generate artificial neuronal activities through Monte Carlo simulation to validate how well the inferred structure describes the true model by comparing $\boldsymbol{C}$ and $\boldsymbol{D}$ with the true values, as shown in Fig. S4D and E respectively. We can see that in general, the predicted values closely align with the true values for all methods, for both $\boldsymbol{C}$ and $\boldsymbol{D}$. First of all, we can see that MLE has the best performance as anticipated, similar to that we observe in the pattering substrates system. This is because there are no restrictions and prior knowledge provided to the inference algorithm. Our GML algorithm performs very well in activity predictions and is better than MAP, which is indeed the GML with one iteration. This suggests that GML as the full evidence-based approach can improve the inference of neuronal activities by optimizing the values of hyperparameters.

In general, by studying the homogeneous *in silico* model, we show that GML performs very well in identifying neuronal types

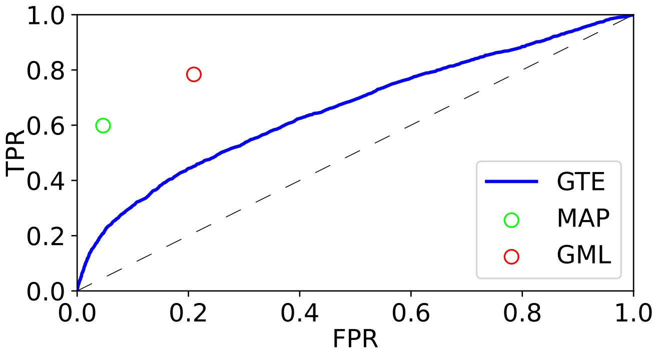

Fig. S5. The receiver operating characteristic (ROC) curve plots the true positive rate (TPR) against the false positive rate (FPR) of identifying effective links for *in silico* experiments using generalized transfer entropy (GTE). The TPR and TFR using our MAP and GML are marked as green and red nodes respectively.

and link existence inference as well as the neuronal activities prediction, even without the constraints imposed by patterned substrates.

C. Sensitivity analysis - *In silico* homogeneous network . To further explore the performance of our algorithms, we conduct a sensitivity analysis on *in silico* neuronal networks with homogeneous connectivity. The synaptic strengths follow the same distributions as described in the main text, with the corresponding means $(\Omega_E, \Omega_I) = (6, 12)$ for strong couplings and $(\Omega_E, \Omega_I) = (4, 8)$ for weak couplings. The analysis is performed on homogeneous networks with $N = 156$ neurons, where each point is averaged over 5 samples.

In the homogeneous network setting, we increase the degree-connectivity by incrementally allowing for longer axon lengths. Starting from having no connections between neurons, we gradually grow the axons and increase the connectivity. At each level of degree connectivity, we take snapshots of the network structure and generate neuronal activities. This allows us to study the performance of our methods as the network connectivity increases.

We observe that MAP consistently outperforms GTE for both weak and strong synaptic strengths in homogeneous networks. This is because as the axons grow and form more connections, the degree-connectivity increases, leading to more active neuronal activity. This provides a richer dataset for inference, allowing MAP to perform structural inference more effectively.

As shown in Fig. S6A, MAP achieves very high accuracy in neuronal type classification, significantly outperforming biased random guessing. This consistent performance highlights the robustness of MAP in classifying neuronal types across different synaptic strengths.

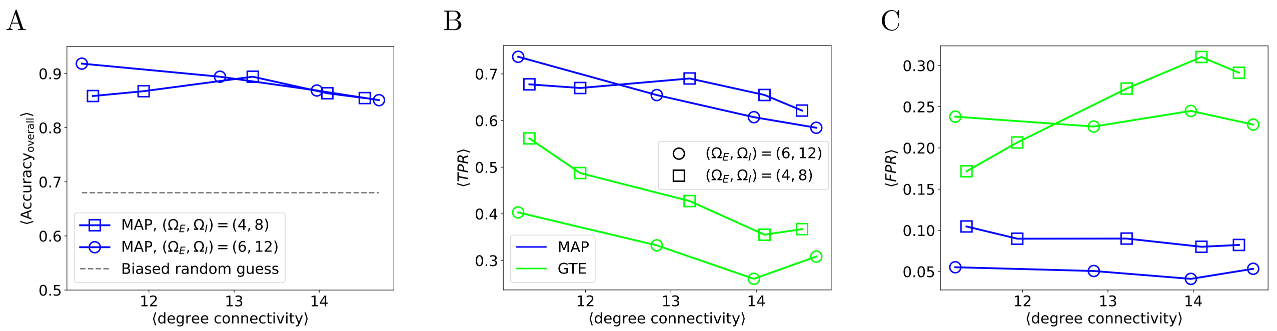

Fig. S6. Sensitivity analysis of MAP and GTE on structural inference for *in silico* homogeneous neuronal networks with different synaptic weight distributions, varying over the average degree-connectivity. A: Average overall performance in neuronal type classification, compared to biased random guessing. B: TPR of identifying effective links. The TPR of GTE is obtained from the ROC curve by fixing the FPR at the same value as MAP. C: FPR of identifying effective links. The FPR of GTE is obtained from the ROC curve by fixing the TPR at the same level as MAP. Results are averaged over 5 samples for a network of $N = 156$ neurons.

In Fig. S6B and C, we observe that MAP has consistently a higher TPR and lower FPR than GTE for identifying effective links, regardless of synaptic strengths. This demonstrates the advantage of MAP in homogeneous networks, where the increased degree-connectivity results in higher neuronal activity, allowing for more reliable inference of effective connections.

For evaluating the dependence of correlation predictability on degree-connectivity, Fig. S7 shows that MLE consistently outperforms MAP in homogeneous networks, which aligns with the general understanding that MLE provides the most accurate parametric model of the data when enough information is available. The higher degree of connectivity in homogeneous networks results in higher neuronal activity, providing sufficient data for MLE to find an optimal solution. However, for the weak synaptic strength case, the advantage of MLE is smaller. This is because of the lower activity level in weakly connected networks, meaning that the data barely provide enough information for obtaining a good solution.

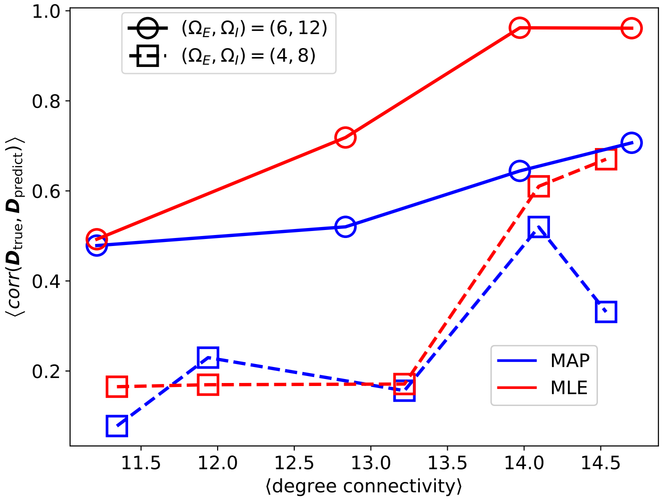

Fig. S7. Sensitivity analysis of performance predictability on *in silico* homogeneous neuronal networks using MAP and MLE. The average correlation between predicted and true delayed-time covariance $\boldsymbol{D}$, for strong and weak synaptic strengths, is shown as a function of the average degree-connectivity. Results are averaged over 5 samples for a network of $N = 156$ neurons.

Although MLE achieves better predictability performance, it ignores real neuronal culture constraints as it cannot provide neuronal type classification or effective connection identification. Our MAP method performs exceptionally well in these tasks and is therefore more suitable for both structural and dynamical inference in neuronal networks.

5. Computational Specifications

In our experiments, we used a standard laptop without GPU acceleration—specifically, an Apple MacBook Pro (late 2023) model. For a system size of $N = 156$, the RAM usage remained under 500MB. The MAP inference was completed in approximately 1 hour, whereas the GML method took around 24 hours. Our observations indicate that the number of time steps required for convergence is consistent across different network sizes. The computational complexity of our methods depends on the number of variables and time steps in the dataset, leading to an overall complexity of $O(TN^2)$, where T represents the time window. Similar methods, such as the pairwise maximum entropy model (MEM), have been shown to operate effectively on systems of comparable size (around $N = 100$) (7). This suggests that our approach is well within practical computational limits, especially given that MEM assumes equilibrium, whereas our methods are suitable for both equilibrium and non-equilibrium systems under certain assumptions.

References

1. Y Roudi, J Hertz, Mean field theory for nonequilibrium network reconstruction. *Phys. review letters* **106**, 048702 (2011).
2. Y Terada, T Obuchi, T Isomura, Y Kabashima, Objective and efficient inference for couplings in neuronal networks. *Adv. neural information processing systems* **31** (2018).
3. M Mézard, J Sakellariou, Exact mean-field inference in asymmetric kinetic ising systems. *J. Stat. Mech. Theory Exp.* **2011**, L07001 (2011).
4. P Zhang, Inference of kinetic ising model on sparse graphs. *J. Stat. Phys.* **148**, 502–512 (2012).
5. R Brout, H Thomas, Molecular field theory, the onsager reaction field and the spherical model. *Phys. Physique Fizika* **3**, 317 (1967).
6. M Neumann, O Steinhauser, The influence of boundary conditions used in machine simulations on the structure of polar systems. *Mol. Phys.* **39**, 437–454 (1980).
7. VK Olsen, JR Whitlock, Y Roudi, The quality and complexity of pairwise maximum entropy models for large cortical populations. *PLOS Comput. Biol.* **20**, 1–30 (2024).
8. W Song, I Cajigas, EN Brown, SF Giszter, Adaptation to elastic loads and bmi robot controls during rat locomotion examined with point-process glms. *Front. Syst. Neurosci.* **9** (2015).
9. JG Orlandi, O Stetter, J Soriano, T Geisel, D Battaglia, Transfer entropy reconstruction and labeling of neuronal connections from simulated calcium imaging. *PloS one* **9**, e98842 (2014).
